# Co-Expression of Adaptor Protein FAM159B with Different Markers for Neuroendocrine Cells: An Immunocytochemical and Immunohistochemical Study

**DOI:** 10.3390/ijms232113503

**Published:** 2022-11-04

**Authors:** Anna-Sophia Liselott Beyer, Daniel Kaemmerer, Jörg Sänger, Amelie Lupp

**Affiliations:** 1 Institute of Pharmacology and Toxicology, Jena University Hospital, Friedrich Schiller University, 07747 Jena, Germany; 2 Department of General and Visceral Surgery, Zentralklinik Bad Berka, 99438 Bad Berka, Germany; 3 Laboratory of Pathology and Cytology Bad Berka, 99438 Bad Berka, Germany

**Keywords:** FAM159B, adaptor protein, neuroendocrine marker, somatostatin receptor, dopamine receptor 2, RGS9, antibody, immunocytochemistry, immunohistochemistry

## Abstract

Little is known about the adaptor protein FAM159B. Recently, FAM159B was shown to be particularly expressed in neuroendocrine cells and tissues, such as pancreatic islets and neuroendocrine cells of the bronchopulmonary and gastrointestinal tracts, as well as in different types of neuroendocrine tumours. To gain insights into possible interactions of FAM159B with other proteins and/or receptors, we analysed the co-expression of FAM159B and various neuroendocrine-specific markers in the cancer cell lines BON-1, PC-3, NCI-h82, OH-1, and A431 and also in human pancreatic tissues and pancreatic neuroendocrine tumours. The markers included prominent markers of neuroendocrine differentiation, such as chromogranin A (CgA), neuron-specific enolase (NSE), synaptophysin (SYP), insulinoma-associated protein 1 (INSM1), neural cell adhesion molecule 1 (NCAM1), serotonin (5-HT), somatostatin-14/28 (SST), and several receptors that are typically expressed by neuroendocrine cells, such as dopamine receptor 2 (D2R), somatostatin receptor (SSTR) 1, 2, 3, 4 and 5, and regulator of G-protein signalling 9 (RGS9). FAM159B was expressed evenly throughout the cytosol in all five cancer cell lines. Immunocytochemical and immunohistochemical analyses revealed co-expression of FAM159B with SYP, INSM1, RGS9, D2R, SSTR2, SSTR3, SSTR4, and SSTR5 and strong overlapping co-localisation with NSE. Double-labelling and co-immunoprecipitation Western blot analyses confirmed a direct association between FAM159B and NSE. These results suggest the involvement of FAM159B in several intracellular signalling pathways and a direct or indirect influence on diverse membrane proteins and receptors.

## 1. Introduction

Adaptor proteins play essential and diverse roles in various cell signalling pathways. They are usually non-enzymatic and regulate different aspects of cell surface-receptor functions through protein–membrane or protein–protein interactions mediated by their modular domains and/or peptide motifs. The β-arrestins are a well-known example of adaptor proteins that play a role in regulating G-protein-coupled receptor signalling.

The adaptor protein FAM159B is a member of the Shisa-like protein family. Shisa-like proteins are closely related to the Shisa family of single-transmembrane proteins; however, the Shisa proteins have an N-terminal domain with six conserved cysteines, whereas the Shisa-like proteins have an N-terminal domain with eight conserved cysteines. Vertebrates have two copies of FAM159: FAM159A and FAM159B. It has been proposed that FAM159B is a transmembrane adaptor that regulates other transmembrane proteins and receptors [1], although the exact function of FAM159B is still unknown.

FAM159B expression has been demonstrated in pancreatic islets and neuroendocrine cells of the stomach mucosa [2] and the presence of FAM159B has been linked to pancreatic beta cell exocytosis [3] and maturation [4]. Using a well-characterised antibody, we recently showed expression of FAM159B not only in pancreatic islets and neuroendocrine cells of the bronchopulmonary and gastrointestinal tracts, but also in different neuronal tissues, bronchial and intestinal epithelia, the epithelium of the larger bile ducts of the liver and gallbladder, the mesangial cells of the glomeruli, the visceral and parietal layer of Bowman’s capsule and the distal tubules of the kidney as well as the syncytiotrophoblasts of the placenta [5]. We also demonstrated expression of FAM159B in many tumour cell lines and tumour entities, with especially high levels in pituitary adenomas, medullary and anaplastic thyroid carcinomas, parathyroid adenomas, lung and ovarian carcinomas, lymphomas, and neuroendocrine tumours of various origins. A subsequent, more in-depth examination of neuroendocrine tumours revealed a correlation between the expression levels of FAM159B and those of various markers commonly expressed in neuroendocrine tumours [5].

In the present study, to gain deeper insight into possible direct or indirect interactions of FAM159B with other proteins and receptors, we performed immunocytochemical and immunohistochemical double-labelling experiments to evaluate FAM159B co-expression with the following markers of neuronal and neuroendocrine tissues: chromogranin A (CgA), neuron-specific enolase (NSE), synaptophysin (SYP), insulinoma-associated protein 1 (INSM1), neural cell adhesion molecule 1 (NCAM1), serotonin (5-HT), somatostatin-14/28 (SST), regulator of G-protein signalling 9 (RGS9), dopamine receptor 2 (D2R), and the somatostatin receptors (SSTR) 1, 2, 3, 4, and 5. Because our previous experiments showed a strong FAM159B immunosignal in the neuroendocrine tumour cell line BON-1 [5], we used BON-1 cells in the present investigation too. We additionally used the prostate cancer cell line PC-3, because of its capacity for neuroendocrine differentiation, and two small cell lung cancer (SCLC) cell lines, NCI-h82 and OH-1, because of their neuroendocrine origin. The epidermoid cell line A431 served as a (negative) control. We complemented these immunocytochemical experiments with corresponding immunohistochemical experiments using tissue samples from the human pancreas and pancreatic neuroendocrine tumours (PanNETs).

## 2. Results

### 2.1. Verification of FAM159B Expression in the Selected Cancer Cell Lines

**Figure 1:**
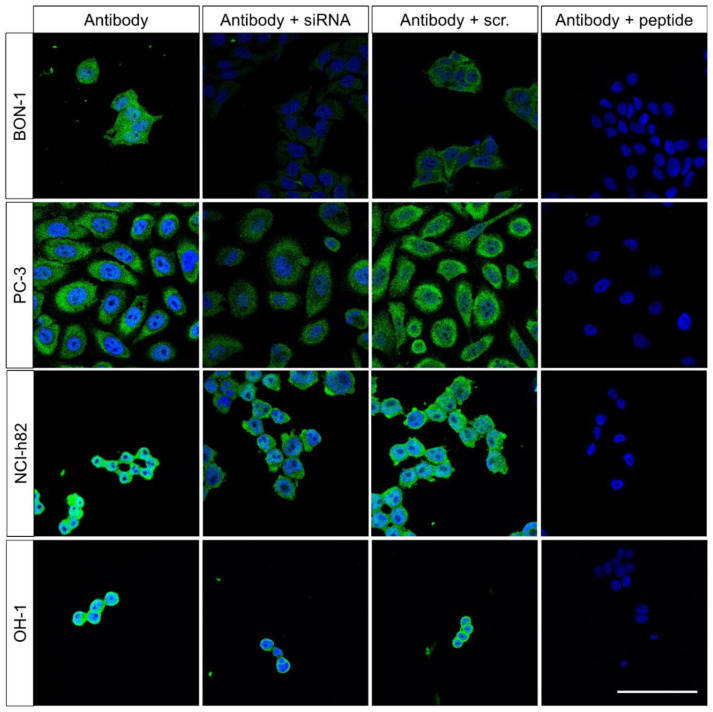
Immunocytochemical analysis of FAM159B expression in BON-1, PC-3, NCI-h82 and OH-1 cells. Left three panels: cells remained untreated or were transfected with specific siRNA or a scrambled siRNA (scr.) and then fixed and stained with anti-FAM159B HPA011778, followed by Alexa Fluor 488-conjugated anti-rabbit secondary antibody. Right panel: For adsorption controls, anti-FAM159B HPA011778 was pre-incubated for 2 h prior to immunocytochemistry with 10 µg/mL of the peptide used to immunise rabbits. Green: immunosignal; blue: DAPI staining of DNA. All photomicrographs were taken at the same magnification. Scale bar: 100 µm.

To verify FAM159B expression in the selected cancer cell lines, and to demonstrate the specificity of the immunosignal obtained in these cells using the antibody HPA011778, we first performed immunocytochemical analyses in untreated cells and cells transfected with a FAM159B-specific siRNA. These experiments revealed a bright immunosignal dispersed throughout the cytosol in untreated BON-1, PC-3, NCI-h82, and OH-1 cells (Figure 1, left panel). In contrast to the other cell lines, A431 cells displayed only very weak FAM159B expression. After transfection of the cancer cell lines with the FAM159B-specific siRNA, the signal intensity was substantially diminished, whereas cells transfected with a scrambled control siRNA displayed a strong immunosignal similar to that in untreated cells (Figure 1, middle two panels). To further check the specificity of the HPA011778 antibody, we performed pre-adsorption experiments with the peptide used to immunise rabbits. When the HPA011778 antibody was incubated with the immunising peptide prior to immunostaining of cells, the immunosignal was completely extinguished in all cases (Figure 1, right panel).

The specificity of HPA011778 was further tested with Western blot analyses. When the cytosolic fraction (“supernatant”) of endogenously FAM159B-expressing BON-1, PC-3, NCI-h82, or OH-1 cells was separated through electrophoresis and immunoblotted, the antibody recognised a band at approximately M_r_ = 16–18 kDa (Supplemental Figure S1, lanes A; for Western blot experiments on BON-1 cells, see [5]). In contrast, no immunosignal could be detected in the wheat germ agarose (WGA) bead fraction, which contained the enriched glycosylated proteins (Supplemental Figure S1, lanes D; [5]). After treatment of the cells with a targeted siRNA, the immunosignal in the cytosolic fraction was diminished (Supplemental Figure S1, lanes B; [5]). To further confirm the specificity of the antibody, it was pre-adsorbed with its immunising peptide and then used for Western blot analysis, which revealed that the immunosignal was completely abolished (Supplemental Figure S1, lanes C; [5]).

### 2.2. Immunocytochemical and Immunohistochemical Double-Labelling Experiments with Different Markers for Neuroendocrine Cells

Using the four FAM159B-expressing cancer cell lines, we performed immunocytochemical double-labelling experiments with several markers that are typically expressed in neuroendocrine cells. We complemented these investigations with corresponding immunohistochemical staining in human pancreas and PanNET samples. In Figure 2, Figure 3 and Figure 4, the merged immunocytochemistry images are shown, and in Figure 5, the merged immunohistochemistry pictures are shown; the complete illustrations with the additional depiction of the individual channels are included in the Supplements (immunocytochemistry experiments: Supplemental Figures S2–S13; immunohistochemistry experiments: Supplemental Figures S14–S20).

**Figure 2:**
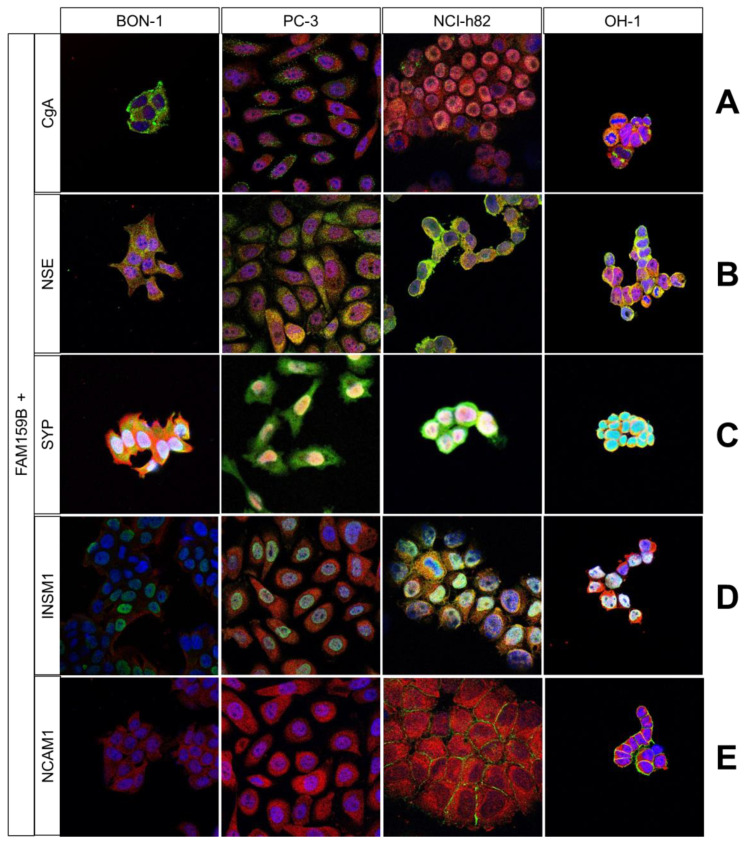
Double-labelling immunocytochemical analysis of FAM159B and chromogranin A (CgA; (**A**)), neuron specific enolase (NSE; (**B**)), synaptophysin (SYP; (**C**)), insulinoma-associated protein 1 (INSM1; (**D**)), or neural cell adhesion molecule 1 (NCAM1; (**E**)) expression in BON-1, PC-3, NCI-h82, and OH-1 cells. (**A**,**B**,**D**,**E**): Labelling for FAM159B was visualised using Cy3-conjugated anti-rabbit antibody (red). Labelling for CgA, NSE, INSM1, and NCAM1 was visualised using Alexa Fluor 488-conjugated anti-mouse antibody (green). (**C**): Labelling for FAM159B was visualised using FITC-conjugated anti-rabbit antibody (green). Labelling for synaptophysin was visualised using Cy3-conjugated anti-rabbit antibody (red). Overlapping expression is shown in orange/yellow colour. All photomicrographs were taken at the same magnification. Scale bar: 100 µm.

FAM159B and CgA displayed slight co-localisation in NCI-h82 and OH-1 cells, whereas they had more separated localisations in BON-1 and PC-3 cells (Figure 2A). The CgA was located in many small, well-defined vesicles in the cytosol, whereas FAM159B was distributed more evenly throughout the cells. The immunohistochemical analyses revealed a partial overlap of the two proteins in pancreatic islets and PanNET samples (Figure 5A). In the islets, the expression of CgA was more prominent in cells located in the periphery than in in the centre of the islets. In the PanNET samples, the intensity and localisation of FAM159B expression were similar to those of CgA expression.

FAM159B and NSE had an abundant and homogeneous distribution throughout the cytosol in all four cancer cell lines (Figure 2B). FAM159B and NSE also showed strong overlap in pancreatic islets and PanNET samples (Figure 5B).

FAM159B and SYP showed extensive co-localisation throughout all four examined cell lines (Figure 2C). They also exhibited a strong overlap in pancreatic islets and PanNET samples (Figure 5C).

FAM159B and INSM1 displayed strong co-localisation in NCI-h82 cells and moderate co-localisation in PC-3 cells (Figure 2D); however, there was no co-localisation of FAM159B and INSM1 in pancreatic tissues or the PanNET samples (Figure 5D). INSM1 was mostly located in and around the nucleus of cells, but it was also present within the cytosol.

**Figure 3:**
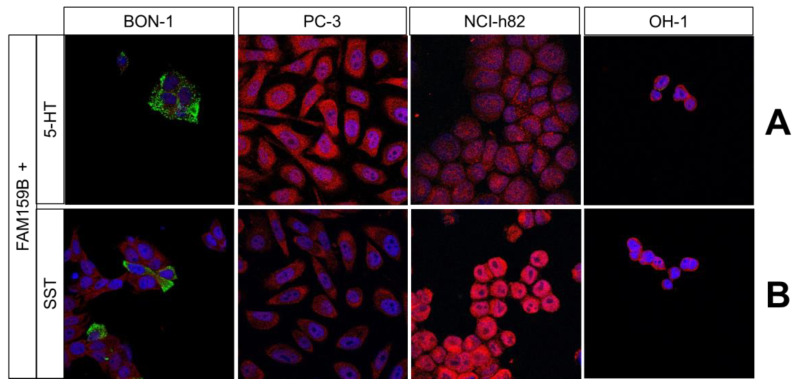
Double-labelling immunocytochemical analysis of FAM159B and serotonin (5-HT; (**A**)) or somatostatin-14/28 (SST; (**B**)) expression in BON-1, PC-3, NCI-h82, and OH-1 cells. Labelling for FAM159B was visualised using Cy3-conjugated anti-rabbit antibody (red). Labelling for 5-HT and SST was visualised using Alexa Fluor 488-conjugated anti-mouse antibody (green). Overlapping expression is shown in orange/yellow colour. All photomicrographs were taken at the same magnification. Scale bar: 100 µm.

FAM159B did not co-localise with NCAM1, 5-HT, or somatostatin-14/28 in any of the cancer cell lines (Figure 2E and Figure 3A,B). Each marker had a distinct localisation, and each was absent from at least one of the cell lines. For example, somatostatin-14/28 was present in BON-1 cells but not in PC-3, NCI-h82, or OH-1 cells (Figure 3B). NCAM1 (Figure 5E) and 5-HT (Figure 5F) showed little to no expression in the pancreatic tissues and PanNET samples. Expression of somatostatin-14/28 (Figure 5G) was limited to specific, single cells within pancreatic islets and PanNETs and exhibited partial overlap with FAM159B within those cells.

**Figure 4:**
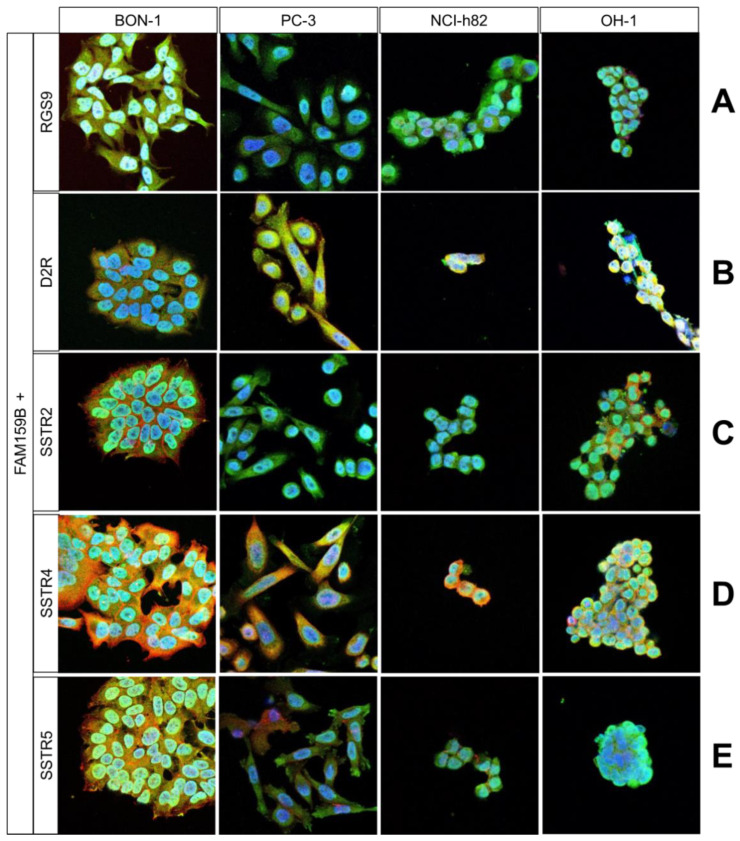
Double-labelling immunocytochemical analysis of FAM159B and regulator of G-protein signalling (RGS9; (**A**)), dopamine receptor 2 (D2R; (**B**)), somatostatin receptor 2 (SSTR2; (**C**)), somatostatin receptor 4 (SSTR4; (**D**)), or somatostatin receptor 5 (SSTR5; (**E**)), expression in BON-1, PC-3, NCI-h82, and OH-1 cells. Labelling for FAM159B was visualised using FITC-conjugated anti-rabbit antibody (green). Labelling for RGS9, D2R, SSTR2, SSTR4, and SSTR5 was visualised using Cy3-conjugated anti-rabbit antibody (red). Overlapping expression is shown in orange/yellow colour. All photomicrographs were taken at the same magnification. Scale bar: 100 µm.

**Figure 5:**
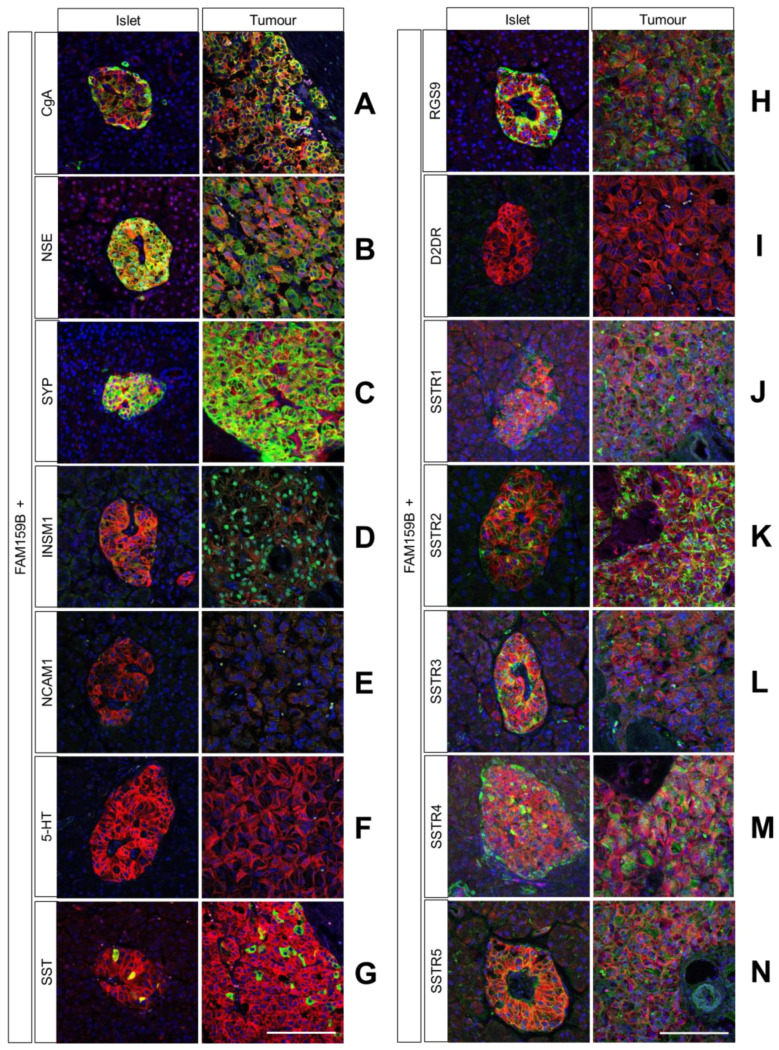
Double-labelling immunohistochemical analysis of FAM159B and chromogranin A (CgA; (**A**)), neuron specific enolase (NSE; (**B**)), synaptophysin (SYP; (**C**)), insulinoma-associated protein 1 (INSM1; (**D**)), neural cell adhesion molecule 1 (NCAM1; (**E**)), serotonin (5-HT; (**F**)), somatostatin-14/28 (SST; (**G**)), regulator of G-protein signalling (RGS9; (**H**)), dopamine receptor 2 (D2R; (**I**)), somatostatin receptor 1 (SSTR1; (**J**)), somatostatin receptor 2 (SSTR2; (**K**)), somatostatin receptor 3 (SSTR3; (**L**)), somatostatin receptor 4 (SSTR4; (**M**)), or somatostatin receptor 5 (SSTR5; (**N**)) expression in human pancreas or pancreatic neuroendocrine tumour tissues. Sections were dewaxed, microwaved in citric acid and incubated with corresponding primary antibodies. Labelling for FAM159B was visualised using Cy3-conjugated anti-rabbit antibody (red). Labelling for CgA, NSE, SYP, INSM1, NCAM1, 5-HT, SST, RGS9, D2R, SSTR1, SSTR2, SSTR3, SSTR4, or SSTR5 was visualised using Alexa Fluor 488-conjugated anti-mouse, anti-rat or anti-rabbit antibody (green). Overlapping expression is shown in orange/yellow colour. All photomicrographs were taken at the same magnification. Scale bar: 100 µm.

FAM159B co-localised with RGS9, D2R, SSTR4, and SSTR5 in the four cancer cell lines to a different extent (Figure 4A–E). Each marker showed an expression pattern similar to that of FAM159B, although SSTR4 additionally displayed significant membranous expression. On the other hand, FAM159B and SSTR2 had distinct localisations within the cell lines with no significant co-localisation. SSTR2 was situated along the membrane, whereas FAM159B was evenly distributed throughout the cytosol. SSTR1 and SSTR3 were not expressed to a significant degree in any of the cell lines investigated. Immunohistochemical analyses revealed that FAM159B was co-expressed with RGS9 (Figure 5H), SSTR2 (Figure 5K), SSTR3 (Figure 5L), SSTR4 (Figure 5M), and SSTR5 (Figure 5N) in pancreatic islets and to a lesser extent in PanNET samples. No expression of D2R (Figure 5I) was observed in the pancreatic islets or PanNET samples. Expression of SSTR1 (Figure 5J) was weak and did not seem to co-localise with the expression of FAM159B in pancreatic islets and PanNET samples.

### 2.3. FAM159B-NSE Co-Localization and Interaction Experiments

**Figure 6:**
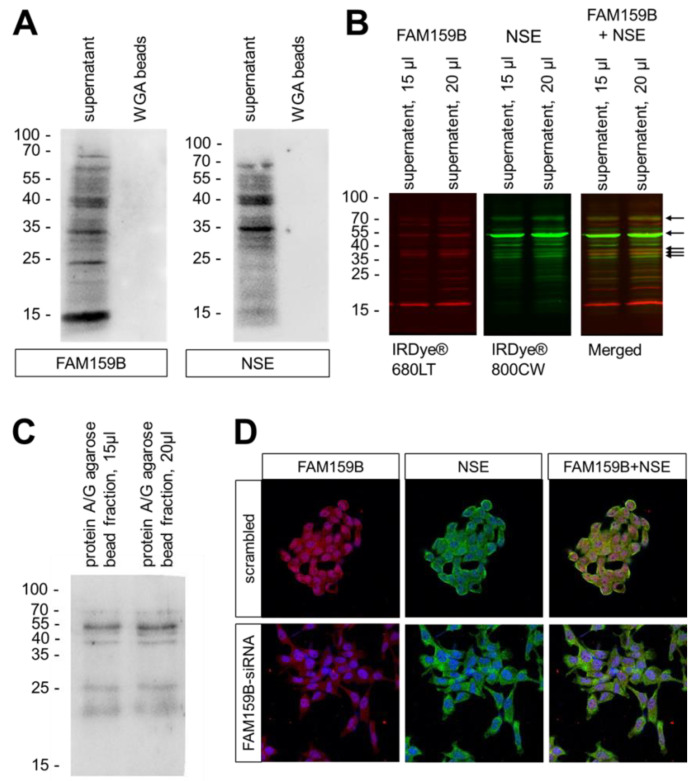
Association between FAM159B and neuron specific enolase (NSE) in BON-1 cells. (**A**) Western blot analysis of the cytosolic fraction (supernatant) and membrane preparations (WGA beads) of BON-1 cells endogenously expressing FAM159B and NSE. The ordinate shows migration of protein molecular-weight markers (kDa). Representative results from one of three independent experiments are shown. (**B**) Double-labelling Western blot analysis of the cytosolic fraction of FAM159B and NSE in BON-1 cells endogenously expressing FAM159B and NSE. FAM159B was visualised using IRDye^®^ 680 LT goat anti-rabbit IgG secondary antibody (red). NSE was visualised using IRDye^®^ 800 CW goat anti-mouse IgG secondary antibody (green). Overlapping bands (Merged) appear in orange/yellow colour (arrows). The ordinate shows migration of protein molecular-weight markers (kDa). Representative results from one of three independent experiments are shown. (**C**) Western blot analysis of the cytosolic fraction of BON-1 cells after co-immunoprecipitation of FAM159B and NSE. The ordinate shows migration of protein molecular-weight markers (kDa). Representative results from one of three independent experiments are shown. (**D**) Double-labelling immunocytochemical analysis of FAM159B and NSE after treatment of the cells with a FAM159B-targeting siRNA or a scrambled siRNA. Cells were fixed and stained with anti-FAM159B and anti-NSE antibody. Labelling for FAM159B was visualised using Cy3-conjugated anti-rabbit antibody (red). Labelling for NSE was visualised using Alexa Fluor 488-conjugated anti-mouse antibody (green). Overlapping expression is shown in orange/yellow colour. All photomicrographs were taken at the same magnification. Scale bar: 100 µm.

As the immunocytochemical and immunohistochemical double-labelling experiments showed strong co-localisation of FAM159B with NSE, we performed Western blot analyses of both proteins in the cytosolic (Figure 6A, “supernatant”) and membrane fractions (Figure 6A, “WGA beads”) of whole-cell lysates of BON-1 cells. Staining with anti-NSE antibody produced three prominent signals at approximately M_r_ = 35–40 kDa in the cytosolic fraction. These signals were also found in corresponding Western blots with anti-FAM159B antibody staining. There was no detectable signal for either protein in Western blots of the membrane preparations (non-cytosolic fraction). To confirm these findings, we performed double-labelling with anti-FAM159B and anti-NSE antibodies on Western blots of the cytosolic fraction of BON-1 cells (Figure 6B), which revealed the same three prominent overlapping signals at approximately M_r_ = 35–40 kDa in addition to two more overlapping signals at approximately M_r_ = 55 kDa and M_r_ = 70 kDa. We further performed co-immunoprecipitation experiments of FAM159B and NSE followed by Western blot analysis in BON-1 cells. Here, the same signals emerged as with the double-labelled Western blot (Figure 6C). We then treated the BON-1 cells either with the specific FAM159B-targeting siRNA or with a scrambled siRNA and double-stained the cells with the anti-FAM159B and the anti-NSE antibody. These studies revealed (although FAM159B expression was slightly downregulated) no difference in NSE expression between the cells treated with the specific FAM159B-targeting siRNA and those treated with a scrambled siRNA (Figure 6D).

## 3. Discussion

### 3.1. Verification of FAM159B Expression in the Selected Cancer Cell Lines

The cancer cell lines BON-1, PC-3, NCI-h82, and OH-1 each displayed equally strong immunosignal for FAM159B in untreated cells. The adaptor protein appeared to be evenly dispersed throughout the cytosol of these cells, most likely in distinct vesicles or associated with other proteins and/or receptors.

Our Western blot analyses confirmed the predicted size of FAM159B as Mr = 17 kDa [5,6]. Because FAM159B was selectively detected within the supernatant (cytosolic fraction) of whole-cell preparations, it seems to lack significant glycosylation, at least in endogenously FAM159B-expressing BON-1, PC-3, NCI-h82, or OH-1 cells.

Incubation of the cells for 24 h with a FAM159B-specific siRNA resulted in a reduction of the signal intensity both in the immunocytochemistry experiments and in the Western blot analyses of all cell lines. Besides confirming the specificity of the antibody, this also indicates that the lifespan of FAM159B protein must be longer than 24 h in each of the cell lines. The specificity of the antibody was further demonstrated by pre-adsorption experiments in which in all four cell lines, both in the immunocytochemistry experiments and in the Western blot analyses, the immunosignal was completely extinguished.

### 3.2. Immunocytochemical and Immunohistochemical Double-Labelling Experiments

**Chromogranin A** is a 439 amino-acid glycoprotein that belongs to the group of acidic proteins referred to as chromogranins. CgA is found in the vesicles of neurons and other neuronal cells. Thus, all neuroendocrine tumour entities can secrete CgA, making CgA an essential marker in neuroendocrine tumour diagnosis [7,8,9,10]. Our immunocytochemical and immunohistochemical analyses revealed partial overlap of CgA and FAM159B, indicating that both proteins exist within the same cells but may not be localised within the same compartments. CgA fragments can be found in Western blots at M_r_∼9–85 kDa, with the parent molecule situated at around M_r_∼70–85 kDa [8]. We did not find signals of an appropriate size in our Western blot analysis of FAM159B, so we concluded that CgA is most likely not directly associated with FAM159B. Nonetheless, the partial overlap between FAM159B and CgA in our immunocytochemical and immunohistochemical analyses suggests that FAM159B may be involved in the functions of CgA.

**Neuron-specific enolase** is an enzyme of the glycolytic pathway that is predominantly found in neurons and cells of the neuroendocrine system. NSE is a cytoplasmic enzyme that catalyses the dehydration of 2-phospho-D-glycerate to phosphoenolpyruvate, thus enabling the formation of high-energy compounds such as ATP and NADH. With this function, NSE belongs to an essential metabolic network and therefore plays a role in many physiological, regulatory, and pathophysiological processes [11,12]. Enolases are composed of non-covalently linked subunits (α, β, and γ) that form homodimeric (αα as α-enolase) or heterodimeric (αβ and ββ as β-enolase; γγ and αγ as γ-enolase) isoenzymes. γ-Enolase is commonly referred to as NSE or ENO2. The subunit molecular masses of NSE are approximately M_r_ = 39 kDa (γ-subunit) and M_r_ = 48 kDa (α-subunit). The mass of the dimeric form is around M_r_ = 78 kDa and varies with the subunit combination [12]. NSE appears during neurogenesis, and its expression increases during early neuronal differentiation. Hence, NSE has been proposed to be a good marker for the maturation and differentiation of various neuronal cells [11]. Furthermore, NSE acts as a neurotropic factor that promotes the survival, differentiation, and regeneration of neuronal cells by activating several signalling pathways, such as the mitogen-activated protein kinase (MAPK) and phosphoinositide 3-kinase (PI3K) pathways [12,13]. NSE has been proposed to be involved in cancer cell protection, tumour growth, and cell migration [11,14] and is currently used as a diagnostic and prognostic tumour marker in various cancers [11,14]. Our immunocytochemical and immunohistochemical analyses revealed strong co-localisation of FAM159B and NSE, suggesting a direct association between the two proteins.

**Synaptophysin** is a 38 kDa vesicle glycoprotein discovered in 1985 that plays a role in synaptic signal transmission [15]. It is found in abundance in a wide variety of neuroendocrine cells and their corresponding tumours as well as in all neurons with synaptic vesicles. In neuroendocrine cells, the glycoprotein is diffusely spread throughout the cytoplasm, whereas neurons exhibit a punctuate expression pattern in their corresponding synaptic regions. Synaptophysin immunoreactivity has been observed for alpha- and beta-cells of the pancreatic islets, as well as for other neuroendocrine cells such as adrenal cortical cells and their tumours [16,17,18]. Our immunocytochemical and immunohistochemical analyses revealed an extensive co-localisation of FAM159B and synaptophysin. This underlines the hypothesis of an involvement of FAM159B in the secretory function of, e.g., neurons and (neuro)endocrine cells [5].

**Insulinoma-associated protein 1** is a zinc-finger transcriptional factor expressed almost exclusively in developing embryonic neuroendocrine tissues. Like FAM159B, INSM1 plays an important role in the development of pancreatic and intestinal neuroendocrine cells [19]. INSM1 is also important for the development of adrenal medulla cells and basal neuronal progenitor cells. Although its mRNA levels are significantly reduced in healthy adult tissues, INSM1 is expressed in various neuroendocrine tumours such as insulinomas, pituitary adenomas and medullary thyroid carcinomas, and it is also a specific marker for SCLC [10,19,20,21]. Our immunocytochemical analyses indicated co-localisation of FAM159B and INSM1 in some of the cancer cell lines, but we were unable to detect significant expression of INSM1 in pancreatic tissues and PanNET samples, which is in line with the fact that INSM1 is abundant exclusively in embryonic developing neuroendocrine tissues and certain cancers. INSM1 is approximately 58 kDa in size and is detectable in the nuclear fraction of whole-cell preparations [22]. Accordingly, our immunocytochemical and immunohistochemical investigations revealed nuclear localisation of INSM1, in contrast to the immunosignal of FAM159B, which was evenly spread throughout the cytosol. Therefore, we conclude that INSM1 and FAM159B may be present within the same cells but do not directly associate with one another.

**Neural cell adhesion molecule 1** (also known as CD56) is a glycoprotein belonging to the large superfamily of immunoglobulins. NCAM1 can be found in the central and peripheral nervous system, the hematopoietic and immune system, and various organs including the heart, stomach, pancreas, and adrenal and thyroid glands. NCAM1 plays an important role in cell adhesion, migration, and differentiation [23,24,25]. Our immunocytochemical and immunohistochemical double-labelling experiments revealed no co-localisation of FAM159B and NCAM1, indicating that these molecules are present in some of the same cells but are not directly associated with one another.

**Serotonin** (5-hydroxytryptamine; 5-HT) is a monoamine widely present in nature, ranging from scorpion venom to various fruits. In mammals, 5-HT can be found in the blood, gastrointestinal enterochromaffin cells, and brain and nerve tissues. As a neurotransmitter in the brain, 5-HT is involved in the regulation of various functions such as body temperature, sleep, appetite, memory and learning, mood, and stress response. As a peripheral hormone, 5-HT plays an important role in the regulation of heart rate, gastrointestinal motility, immunity, vascular tone, and platelet function [26,27,28]. Our immunocytochemical and immunohistochemical double-labelling experiments revealed no co-localisation of FAM159B and 5-HT, suggesting that although they are present in some of the same cells, they are not directly associated with one another.

**Somatostatin** (SST) is a peptide hormone produced in the central nervous system and endocrine cells. In mammals, SST originates from pre-prosomatostatin and is later processed into a shorter form (somatostatin-14) and a longer form (somatostatin-28) that are stored in secretory granules. Both peptides play a role in various biological processes, including the regulation of growth hormone and various neurotransmitters and hormones such as insulin and glucagon in the pancreas [29]. Our immunohistochemical and immunocytochemical double-labelling analyses indicated co-expression of FAM159B and somatostatin-14/28 only in a few cells. Because FAM159B and somatostatin-14/28 showed partial overlap when they were present in the same cell, it is plausible that FAM159B participates in somatostatin-14/28 signalling pathways, as somatostatin-14/28 is involved in the regulation of insulin and glucagon synthesis and secretion in the pancreas [29], and FAM159B has been shown to play a role in glucose/insulin homeostasis [3].

**Regulators of G-protein signalling** (RGS) proteins are a family of proteins that accelerate the termination of effector stimulation after G-protein coupled receptor (GPCR) activation. These proteins are multi-functional and may serve as scaffolding proteins, bringing together multiple activators, regulators, and effectors of GPCR function [30,31]. Consequently, interaction of these proteins with RGS proteins affects their localisation, activity, and stability. RGS9 controls fundamental functions such as vision and behaviour and has a distinct regional and cellular distribution [30,31]. The two splice isoforms, RGS9-1 and RGS9-2, differ in the structure of their C-terminus, with RGS9-1 possessing a motif composed of 18 amino acids and RGS9-2 possessing a motif composed of 209 amino acids. RGS9-1 regulates phototransduction in the rods and cones of the eye [30]. RGS9-2 has a molecular weight of 76 kDa and regulates dopamine and opioid signalling in basal ganglia as well as reward behaviour and movement coordination through effects on D2 dopamine and µ-opioid receptor (MOR) signalling [31,32,33,34,35]. Thus, RGS9-2 regulates analgesic tolerance and is highly enriched in MOR-expressing cells, the striatum, and, to a lesser degree, the periaqueductal grey and spinal cord [32,33,34,35]. Furthermore, depending on the agonist administered, RGS9-2 is a positive or negative modulator of MOR-mediated behavioural response in mice. These effects are the result of agonist-dependent complex formation with other signal transduction partners in the striatum [32]. Overall, current evidence points to a model in which RGS proteins function as part of a larger signalling complex including various receptors, effector enzymes, scaffolding, and other signalling proteins [31]. We evaluated a possible co-expression of FAM159B with RGS9, because in the few studies available so far on FAM159B expression, RGS9 was explicitly mentioned along with FAM159B. Like FAM159B, RGS9 has been demonstrated to be selectively expressed in pancreatic islet cells [2] and, like FAM159B, it has been shown to represent a candidate regulator of physiological beta cell function and to be positively correlated with beta cell exocytosis [3].

The neurotransmitter **dopamine** is produced in dopaminergic neurons by successive hydroxylation and decarboxylation of tyrosine. Dopamine plays a crucial role during neuronal proliferation and differentiation in the central nervous system of adults. The corresponding dopamine receptors (DRs) belong to the large group of seven-transmembrane GPCRs and can be divided into two subtypes: D1R-like receptors (D1R and D5R) and D2R-like receptors (D2R, D3R, and D4R) [36]. D2R-like receptors are mainly expressed in the striatum, external globus pallidus, core of the nucleus accumbens, amygdala, cerebral cortex, hippocampus, and pituitary. Because of alternate splicing, D2R exists in two isoforms [D2S (short) and D2L (long)] that differ in the presence of an additional 29 amino acids on the third intracellular loop of D2L, which are absent in D2S [36]. Activation of D2R inhibits adenylyl cyclase and, thus, cAMP production as well as protein kinase A (PKA) activity. Downstream signalling following D2R activation activates cell proliferation-related pathways such as the MAPK and protein kinase B (AKT) pathways. DRs can signal via classic GPCR signalling mechanisms or through alternative signalling pathways involving receptor tyrosine kinases, ion channels, or protein–protein interactions with β-arrestins [36]. Overall, dopamine and its receptors are involved in the regulation of motor activity and are of importance in several neurological disorders such as Parkinson’s disease, schizophrenia, and bipolar disorder [36,37,38].

**Somatostatin receptors** (SSTRs) belong to the large family of GPCRs and are activated by SST or its synthetic analogues. SSTRs are based in cellular membranes and are connected to transmembrane K^+^ channels, Ca^2+^ channels, and various intracellular enzymes, including adenylate cyclase, phosphotyrosine phosphatases, and others. In total, five SSTR subtypes (SSTR1, 2, 3, 4, and 5) have been identified and shown to be expressed in numerous healthy tissues and tumours. Following activation, SSTRs and their downstream signalling cascades lead to antisecretory effects, suppression of tumour cell proliferation and survival, and inhibition of angiogenesis. Overall, SSTRs are involved in a vast number of signalling pathways and exert receptor- and/or tissue-specific effects following their activation by SST or SST analogues [39,40,41].

Our immunocytochemical and immunohistochemical analyses revealed extensive co-localisation of FAM159B with RGS9, D2R, SSTR2, SSTR3, SSTR4, and SSTR5. A specific interaction between RGS9 and D2R that accelerates the termination of D2R signals was observed previously and hypothesised to be mediated by a third-party protein [42]. D2R and RGS9-2 expression and interaction have also been observed in the striatum [35], where corresponding FAM159B expression has also been detected. Additionally, our previous study showed a significant positive correlation between FAM159B and D2R expression in neuroendocrine neoplasms [5]. As our present analyses show that FAM159 expression co-localised with D2R and RGS9 expression, it is possible that FAM159 is involved in mediating the interaction between RGS9 and D2R. Further studies should aim at a more in-depth analysis of the D2R/RGS9/FAM159B relationship in the striatum and its role in µ-opioid receptor signalling.

Our previous study revealed FAM159B expression in several normal and neoplastic human tissues and identified positive correlations between FAM159B expression and tumour proliferation and histological grade in neuroendocrine neoplasms. We hypothesised that FAM159B may play a direct or indirect role in the secretion of various neurotransmitters and hormones [5]. Because our present results indicate strong co-localisation of FAM159B with SSTR2, SSTR3, SSTR4, SSTR5, and D2R, it is quite possible that FAM159B is involved in the respective downstream signalling pathways of those molecules. This may include the MAPK/ERK pathway, which is essential for forwarding signals from cell surface receptors to the nucleus. The MAPK/ERK pathway regulates the growth, proliferation, differentiation, and survival of many cell types and is de-regulated in several cancers [43]. In addition, the D2R receptor activates the PI3K/AKT pathway [44], which is essential for gene transcription, cell proliferation, and migration. De-regulation of the PI3K/AKT pathway is frequently observed in various cancers and leads to increased proliferation and decreased apoptosis [45]. The involvement of FAM159B in these and other pathways may explain its expression in a large number of normal and neoplastic human tissues as well as its positive correlation with tumour proliferation and histological grade.

### 3.3. Association between FAM159B and NSE

With the FAM159B–NSE double-labelling Western blot analyses and co-immunoprecipitation experiments, we were able to identify some of the additional signals detected by the anti-FAM159B HPA011778 antibody besides the band observed at M_r_ 16–18 kDa, representing the expected molecular weight of FAM159B itself. The bands at M_r_ = 35–40 kDa and the signal at M_r_ = 40–55 kDa possibly represent FAM159B in association with homodimeric or heterodimeric forms of NSE, as we found identical signals in the separate Western blot analyses of FAM159B and NSE, the double-labelled immunoblot and the respective analyses of the FAM159B–NSE co-immunoprecipitation experiments in BON-1 cells. The signals at 35–40 kDa and 40–55 kDa may represent FAM159B in association with γ- and/or α-subunits of NSE, whereas the signal at around 70 kDa may represent FAM159B in association with the dimeric form of NSE. The overlapping signals on Western blots clearly suggest an association between FAM159B and NSE. Both proteins have been implicated in similar processes within living organisms. NSE is involved in the formation of high-energy compounds, providing cells and organisms with some of the most essential components they need for life. FAM159B, on the other hand, plays a role in glucose homeostasis and, potentially, the regulation of insulin exocytosis [3]. It is therefore plausible for FAM159B to be involved in similar, if not the same, biochemical pathways as NSE. Furthermore, FAM159B may mediate protein–protein or protein–membrane interactions and function in the production of high-energy compounds. Both FAM159B and NSE have been described as maturation markers: NSE for neuronal structures and FAM159B for β-cells. It is therefore possible that FAM159B may also be a maturation factor along with NSE in neuronal tissues. Nevertheless, since there was no difference in NSE expression between the cells treated with the specific FAM159B-targeting siRNA and those treated with a scrambled siRNA, FAM159B apparently is not involved in the regulation of NSE expression.

## 4. Materials and Methods

### 4.1. Antibody

The rabbit polyclonal anti-FAM159B antibody (HPA011778) was purchased from Atlas Antibodies AB (Bromma, Sweden). The sequence of the peptide used for rabbit immunisations was as follows: TKPQRLDTGLKLQHLEASSTQEGKSNGKTKALNSNAASNAT NETYYEADDIIQEKTMDATQIHIA. The peptide PrEST Antigen FAM159B (APrEST71583) was also obtained from Atlas Antibodies AB (Bromma, Sweden).

### 4.2. Tumour Cell Lines

The following tumour cell lines were used: the neuroendocrine tumour cell line BON-1, the prostate cancer cell line PC-3, the SCLC cell lines NCI-h82 and OH-1, and the epidermoid cell line A431 (DSMZ, Braunschweig, Germany). BON-1 cells were originally isolated and cultured in 1986 from a lymph node metastasis of a functional neuroendocrine tumour in a male patient. BON-1 cells contain CgA and serotonin secretory granules but do not secrete gastrin, somatostatin, glucagon, insulin, vasoactive intestinal peptide, or pancreatic polypeptide [46]. The PC-3 cell line was established from bone metastasis of a prostate carcinoma of a 62-year-old Caucasian male in 1979. A key characteristic of PC-3 cells is their androgen independency [47]. PC-3 cells express significant amounts of CgA and NSE but do not express androgen receptors or prostate-specific antigens. Amongst the various prostate cancer cell lines that are available, PC-3 represents relatively aggressive and rapidly growing tumours [48]. Furthermore, PC-3 cells display characteristics that place them closer to neuroendocrine carcinomas or small cell carcinomas than to adenocarcinomas of the prostate [49]. The SCLC cell line NCI-h82 was isolated from a tissue sample of a 40-year-old Caucasian male [50]. NCI-h82 cells express only a few neuroendocrine markers and have a short doubling time [51]. OH-1 cells were originally isolated and cultured from a pleural effusion of a 43-year-old male patient in 1980. The cell line is characterised by high L-DOPA-decarboxylase activity [52,53]. The epidermoid cell line A431 originates from the epidermis of an 85-year-old female patient [54,55].

Cells were cultured in DMEM/Hams F12 1:1 (*v*/*v*) (BON-1 cells), RPMI 1640 (PC-3 and NCI-h82 cells), or DMEM (OH-1 cells), supplemented with 10% (*v*/*v*) FCS, 100 IU/mL penicillin, 100 µg/mL streptomycin, and 2 mM glutamine. For the experiments, the cells were grown in 24-well-plates or 75-cm^2^ culture flasks until 80% confluence and then subjected to immunocytochemistry or Western blot analysis.

### 4.3. Immunocytochemistry

Cell lines were grown overnight on glass coverslips to 80% confluence. The cells were then washed with phosphate-buffered saline (PBS) and fixed with 4% paraformaldehyde and 0.2% picric acid in phosphate buffer (pH 6.9) for 20 min at room temperature. After thorough washing with PBS, the cells were incubated with anti-FAM159B HPA011778 antibody (1:100) overnight at 4 °C. The next day, the cells were washed and incubated with Alexa Fluor 488-conjugated secondary antibody (dilution 1:5000; Invitrogen, Karlsruhe, Germany) in darkness for 2 h at room temperature. Samples were then mounted (Invitrogen Fluoromount G, with DAPI; Thermo Fischer Scientific, Waltham, MA, USA) and examined using a Zeiss LSM 510 META laser scanning confocal microscope (Jena, Germany).

When indicated, the endogenous expression of FAM159B in BON-1, PC-3, NCI-h82, or OH-1 cells was silenced for 24 h using a chemically synthesised single-stranded RNA oligonucleotide [ID 264018; Ambion by Life Technologies (Thermo Fischer Scientific), Waltham, MA, USA] according to the manufacturer’s instructions. A scrambled siRNA was used as a negative control (Santa Cruz Biotechnology, Dallas, TX, USA).

For adsorption controls, the anti-FAM159B HPA011778 antibody was pre-incubated for 2 h at room temperature with 10 µg/mL of the peptide PrEST antigen (Atlas Antibodies, Bromma, Sweden) prior to immunocytochemistry.

For double-labelling immunocytochemistry with FAM159B and CgA, NSE, INSM1, NCAM1, serotonin, or SST (antibody detects both SST-14 and SST-28), fixed cells were incubated with anti-FAM159B HPA011778 antibody (1:100) and either anti-CgA, anti-NSE, anti-NCAM1, anti-INSM1, anti-SST, or anti-serotonin antibody overnight at 4 °C (see Table 1 for host species, clonality, clone number, manufacturer, and dilutions). The next day, the cells were washed and incubated in darkness for 2 h at room temperature with Cy3-conjugated anti-rabbit secondary antibody and Alexa Fluor 488-conjugated anti-mouse or anti-rat secondary antibody. Samples were then covered by a cover slip (Fluoromount G, with DAPI; Thermo Fischer Scientific, Waltham, MA, USA) and analysed using a Zeiss LSM 510 META laser scanning confocal microscope (Jena, Germany).

Because the anti-SYP, anti-RGS9, anti-D2R (detects both the short and the long version of the receptor), anti-SST1, anti-SST2, anti-SST3, anti-SST4 and anti-SST5 antibodies, like the anti-FAM159B HPA011778 antibody, are derived from rabbits, the fixed cells were first incubated with the corresponding antibody for the primary receptor or marker overnight at 4 °C. The following day, the cells were washed and incubated for 2 h in darkness at room temperature with Cy3-conjugated anti-rabbit secondary antibody. The cells were then washed thoroughly and incubated overnight at 4 °C with a rabbit anti-FAM159B-FITC-conjugated antibody (LifeSpan BioSciences Inc., Seattle, WA, USA).

### 4.4. Western Blot Analysis

BON-1, PC-3, NCI-h82, or OH-1 cells were seeded into 60-mm petri dishes and grown to 80% confluence. The cells were then lysed in detergent buffer [150 mM NaCl, 50 mM Tris-HCl (pH 7.4), 5 mM EDTA, 1% Triton X-100, 0.5% Na-deoxycholate, 0.1% SDS, 100 mM phenylmethylsulfonylfluoride, 10 mg/mL leupeptin, 5 mg/mL aprotinin, 1 mg/mL pepstatin A]. Next, samples were treated with wheat germ lectin agarose beads. The supernatant and bead fractions were separately subjected to 10% SDS-polyacrylamide gel electrophoresis and immunoblotted onto polyvinylidene fluoride (PVDF) membranes. The blots were then incubated at 4 °C overnight with rabbit polyclonal anti-FAM159B HPA011778 antibody or anti-NSE antibody, followed by incubation for 1.5 h at room temperature with a peroxidase-conjugated secondary anti-rabbit antibody for enhanced chemiluminescence detection (Amersham, Braunschweig, Germany).

When indicated, the endogenous expression of FAM159B in BON-1, PC-3, NCI-h82, or OH-1 cells was silenced for 24 h using a chemically synthesised single-stranded RNA oligonucleotide (see above) according to the manufacturer’s instructions. A scrambled siRNA was used as a negative control (Santa Cruz Biotechnology).

For adsorption controls, the anti-FAM159B HPA011778 antibody was pre-incubated for 2 h at room temperature with 10 µg/mL of the peptide PrEST antigen (Atlas Antibodies) prior to immunoblotting.

For double-labelling Western blot analysis, the blots were incubated with both anti-FAM159B antibody and anti-NSE antibody overnight at 4 °C. The next day, the PVDF membrane was treated with IRDye^®^ 800 CW goat anti-mouse (dilution 1:1000; 926-32210, LI-COR Biosciences, Lincoln, NE, USA) and IRDye^®^ 680 LT goat anti-rabbit (dilution 1:1000; 926-68021, LI-COR Biosciences, Lincoln, NE, USA) secondary antibodies for 30 min in the dark at room temperature. The membrane was then washed and dried and visualised the following day using an Odyssey infrared imager (LI-COR Biosciences).

For co-immunoprecipitation, BON-1 cells were seeded into 60-mm petri dishes and grown to 80% confluence. The cells were then lysed in detergent buffer [150 mM NaCl, 50 mM Tris-HCl (pH 7.4), 5 mM EDTA, 1% Triton X-100, 0.5% Na-deoxycholate, 0.1% SDS, 100 mM phenylmethylsulfonylfluoride, 10 mg/mL leupeptin, 5 mg/mL aprotinin, 1 mg/mL pepstatin A]. Samples were then subjected to untreated protein A/G PLUS agarose beads (sc-2003, Santa Cruz Biotechnology) to bind off any interfering immunoglobulins. After removing the beads, the supernatant was incubated with anti-FAM159B HPA011778 antibody for 1 h at 4 °C. Samples were then subjected to new protein A/G PLUS agarose beads for 1 h at 4 °C, this time with the aim of binding the antibody-coupled FAM159B. The bead fraction was subjected to 10% SDS-polyacrylamide gel electrophoresis and immunoblotted onto polyvinylidene fluoride (PVDF) membranes. The blot was incubated at 4 °C overnight with anti-NSE antibody, followed by incubation for 1.5 h at room temperature with a peroxidase-conjugated secondary anti-rabbit antibody (Santa Cruz Biotechnology) for enhanced chemiluminescence detection (Amersham).

### 4.5. Immunohistochemistry

Completely anonymised, archived, formalin-fixed and paraffin-embedded samples from human pancreas and from pancreatic neuroendocrine tumours (*n* = 3 each) were obtained from the Institute of Pathology and Cytology Bad Berka (Bad Berka, Germany). All procedures performed in this study involving human participants were in accordance with both the ethical standards of the institutional or national research committee and the 1964 Helsinki declaration and its later amendments. Permission for the use of the tissue samples was obtained from the local ethics committee (Ethikkommission der Landesärztekammer Thüringen).

From the paraffin blocks, 4-µm sections were prepared and floated onto positively charged slides. Samples were dewaxed and rehydrated in a descending alcohol series, during which endogenous peroxidase was blocked by incubation of the samples in 0.3% H_2_O_2_ in methanol for 45 min. For antigen retrieval, samples were microwaved in 10 mM citric acid (pH 6.0) for 16 min at 600 W. For double-labelling experiments with antibodies originating from different species, samples were incubated overnight at 4 °C with anti-FAM159B HPA011778 antibody together with antibodies for CgA, NSE, NCAM1, INSM1, SST (detecting both SST-14 and SST-28), or serotonin (see Table 1 for host species, clonality, clone number, manufacturer, and dilutions). Next, the samples were incubated with Cy3-conjugated anti-rabbit secondary antibody and Alexa Fluor 488-conjugated anti-mouse or anti-rat secondary antibody. The samples were then mounted in Invitrogen Fluoromount G with DAPI (Thermo Fisher Scientific, Waltham, MA, USA) and examined using a Zeiss LSM 510 META laser scanning confocal microscope (Jena, Germany).

For double-labelling immunohistochemical experiments using two rabbit primary antibodies, we first tried to perform the experiments using an FITC-conjugated anti-FAM159B primary antibody. This method worked for immunocytochemistry but not for immunohistochemistry, because the obtained signal for anti-FAM159B-FITC in the tissue samples was very weak and differed significantly from that of unconjugated anti-FAM159B. We, therefore, decided to stain two consecutive samples and merge the images digitally. Hence, for double-labelling experiments using rabbit antibodies against SYP, RGS9, D2R (detects both the short and the long version of the receptor), SSTR1, SSTR2, SSTR3, SSTR4, and SSTR5, two consecutive 4-µm sections were prepared from the paraffin blocks, and each was floated onto a positively charged slide. One slide was incubated overnight at 4 °C with anti-FAM159B HPA011778 antibody, while the other was incubated with SYP antibody, RGS9 antibody, D2R antibody, or one of the SSTR antibodies. The following day, the slides were incubated with their respective secondary antibodies: Cy3-conjugated anti-rabbit secondary antibody for the slide previously incubated with anti-FAM159B antibody, and Alexa Fluor 488-conjugated anti-rabbit secondary antibody for the slide previously incubated with the SYP, RGS9, D2R, or SSTR antibody. The slides were then mounted in Invitrogen Fluoromount G with DAPI (Thermo Fisher Scientific, Waltham, MA, USA) and examined using a Zeiss LSM 510 META laser scanning confocal microscope (Jena, Germany). The two separate images were then merged digitally.

## 5. Conclusions

We characterised the expression of the adaptor protein FAM159B alongside that of other neuroendocrine-specific proteins and receptors in the cancer cell lines BON-1, PC-3, NCI-h82, and OH-1 as well as human pancreas and pancreatic neuroendocrine tumour samples. The neuroendocrine-specific markers included CgA, NSE, SYP, NCAM1, INSM1, serotonin, somatostatin 14/28, RGS9, D2R, and SSTR1–5. FAM159B showed strong co-localisation with NSE, but also with SYP, INSM1, RGS9, D2R, SSTR2, SSTR3, SSTR4, and SSTR5. Overall, these results implicate the involvement of FAM159B in a number of signalling pathways and direct or indirect regulation of diverse membrane proteins and receptors. However, more research is needed to clarify the exact role and functions of FAM159B within the human body.

## 6. Limitations

Our current study of the co-expression of FAM159B with different markers for neuroendocrine tumours has some limitations. First, the amount of previous research on FAM159B remains very little and the exact function of the adaptor protein is still unknown. Due to this, this work is more descriptive in nature and does not provide direct answers, but rather aims at setting a starting point or foundation to build upon for further research. Further, this study only examines tissue samples with a human origin and thus results cannot be assumed to be identical within other species (such as rat and mouse). Thus, it would be interesting to see if FAM159B shows similar expression patterns across different species. Finally, this study only examines the co-expression of FAM159B with a total of 14 proteins/receptors. Clearly, there are many other molecules to consider in future research.

## Figures and Tables

**Table 1:** Antibodies used.

Antibody	Clone	Type	Manufacturer	Dilution
FAM159B HPA011778	---	rabbit polyclonal	Atlas Antibodies AB, Bromma, Sweden	1:100
FAM159B-FITC conjugated	---	rabbit polyclonal	Biozol, Eching, Germany	1:100
CgA	LK2H10	mouse monoclonal	BioLogo, Kronshagen, Germany	1:50
NSE	ENO2/1375	mouse monoclonal	Abcam, Cambridge, MA, USA	1:2500
SYP	YE269	rabbit monoclonal	Abcam, Cambridge, MA, USA	1:800
INSM1	A-8	mouse monoclonal	Santa Cruz Biotechnology, Dallas, TX, USA	1:100
NCAM1	123C3	mouse monoclonal	Santa Cruz Biotechnology, Dallas, TX, USA	1:500
Serotonin		mouse	Dako, Glostrup, Denmark	1:50
SST14/28	YC7	rat monoclonal	Abcam, Cambridge, MA, USA	1:300
RGS9	---	rabbit polyclonal	ATLAS Antibodies AB, Bromma Sweden	1:500
D2R	960710	mouse monoclonal	Bio-Techne GmbH, Wiesbaden, Germany	1:100
SSTR1	UMB-7	rabbit monoclonal	Abcam, Cambridge, MA, USA	1:25
SSTR2	UMB-1	rabbit monoclonal	Abcam, Cambridge, MA, USA	1:10
SSTR3	UMB-5	rabbit monoclonal	Abcam, Cambridge, MA, USA	1:20
SSTR4		rabbit monoclonal	Abcam, Cambridge, MA, USA	1:10
SSTR5	UMB-4	rabbit monoclonal	Abcam, Cambridge, MA, USA	1:10
Alexa Fluor 488-conjugated anti-mouse	---	donkey polyclonal	Invitrogen, Karlsruhe, Germany	1:5000
Alexa Fluor 488-conjugated anti-rat	---	donkey polyclonal	Invitrogen, Karlsruhe, Germany	1:5000
Alexa Fluor 488-conjugated anti-rabbit	---	goat polyclonal	Invitrogen, Karlsruhe, Germany	1:5000
Cy3-conjugated anti-rabbit	---t	goat polyclonal	Invitrogen, Karlsruhe, Germany	1:5000
peroxidase-conjugated anti-rabbit	---	goat polyclonal	Santa Cruz Biotechnology, Dallas, TX, USA	1:5000

## Data Availability

The data that support the findings of this study are available from the corresponding author upon reasonable request.

## Supplementary Materials

The following supporting information can be downloaded at: https://www.mdpi.com/article/10.3390/ijms232113503/s1.

## Abbreviation

5-HT	serotonin
CgA	chromogranin A
D2R	dopamine receptor 2
GPCR	G-protein coupled receptor
INSM1	insulinoma-associated protein 1
MOR	µ-opioid receptor
NCAM1	neural cell adhesion molecule 1
NSE	neuron-specific enolase
RGS9	regulator of G-protein signalling 9
SST	somatostatin
SCLC	small-cell lung cancer
SSTR	somatostatin receptor
SYP	synaptophysin
