# Supplementary material for: Co-Expression of Adaptor Protein FAM159B with Different Markers for Neuroendocrine Cells: An Immunocytochemical and Immunohistochemical Study
Source: Int J Mol Sci. 2022 Nov 4;23(21):13503. doi: 10.3390/ijms232113503 (PMC9655893; doi:10.3390/ijms232113503)
Supplement: Supplementary file 1 [file ijms-23-13503-s001.zip › ijms-1867258-supplementary.pdf]

## Supplemental Figure S1

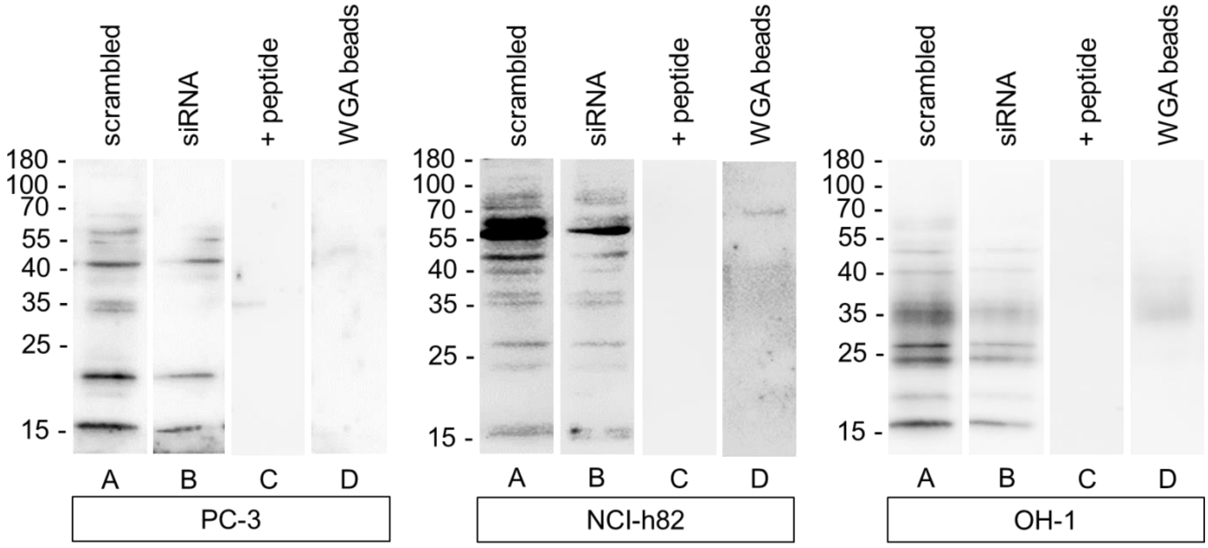


**Supplemental Figure S1: Western blot analysis of FAM159B expression in PC-3, NCI-h82, and OH-1 cells.** Western blot analysis of the supernatant from whole-cell preparations of PC-3, NCI-h82, and OH-1 cells, which endogenously express FAM159B, after transfection of a scrambled siRNA (scrambled) as a negative control (lanes A) or specific siRNA (lanes B). Lanes C: Western blot analysis of the supernatant from whole-cell preparations, when the antibody was pre-adsorbed with the immunising peptide. Lanes D: Western blot analysis of wheat germ agarose (WGA) bead fractions containing glycosylated proteins. Ordinate: migration of protein molecular weight markers (kDa). Representative results from one of three independent experiments are shown.

## Supplemental Figure S2

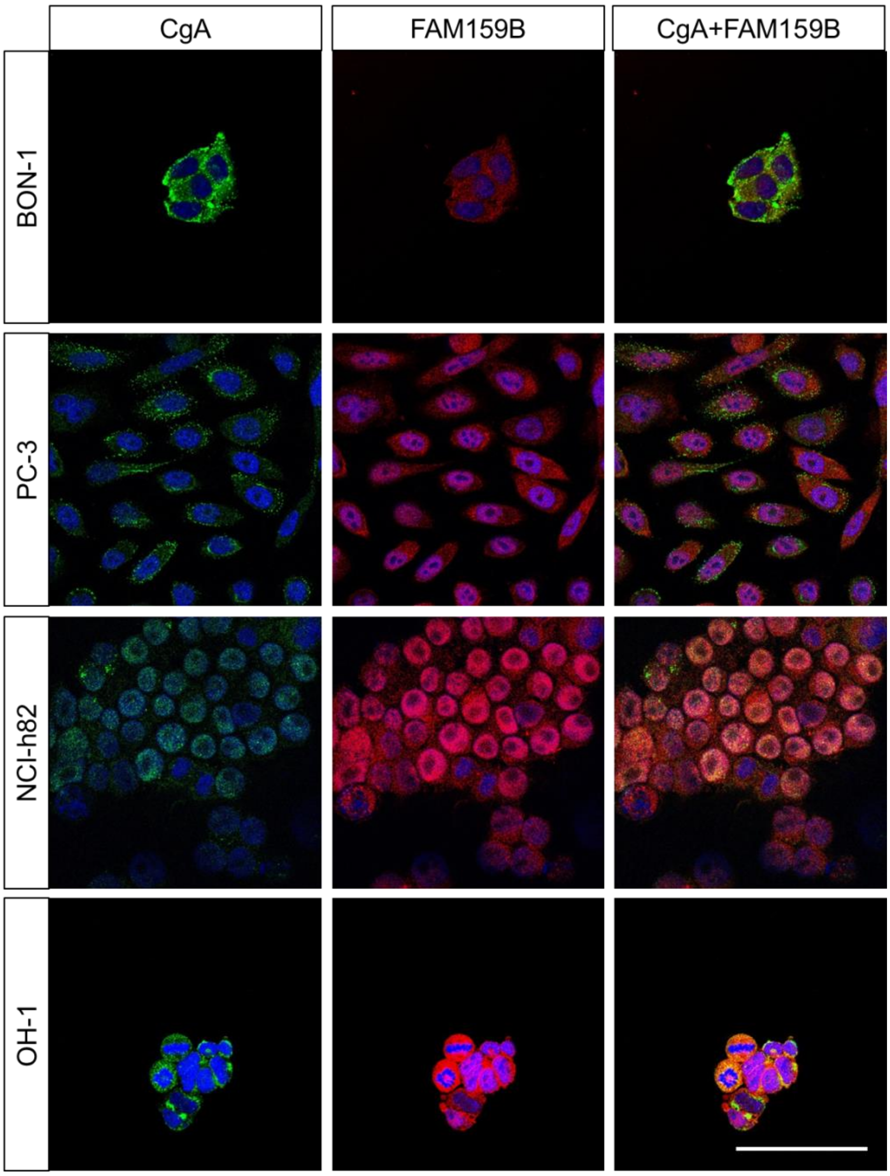


**Supplemental Figure S2: Double-labelling immunocytochemical analysis of FAM159B and chromogranin A (CgA) expression in BON-1, PC-3, NCI-h82, and OH-1 cells.** Labelling for FAM159B was visualised using Cy3-conjugated anti-rabbit antibody (red). Labelling for CgA was visualised using Alexa Fluor 488-conjugated anti-mouse antibody (green). Overlapping expression is shown in orange/yellow colour. All photomicrographs were taken at the same magnification. Scale bar: 100 µm.

## Supplemental Figure S3

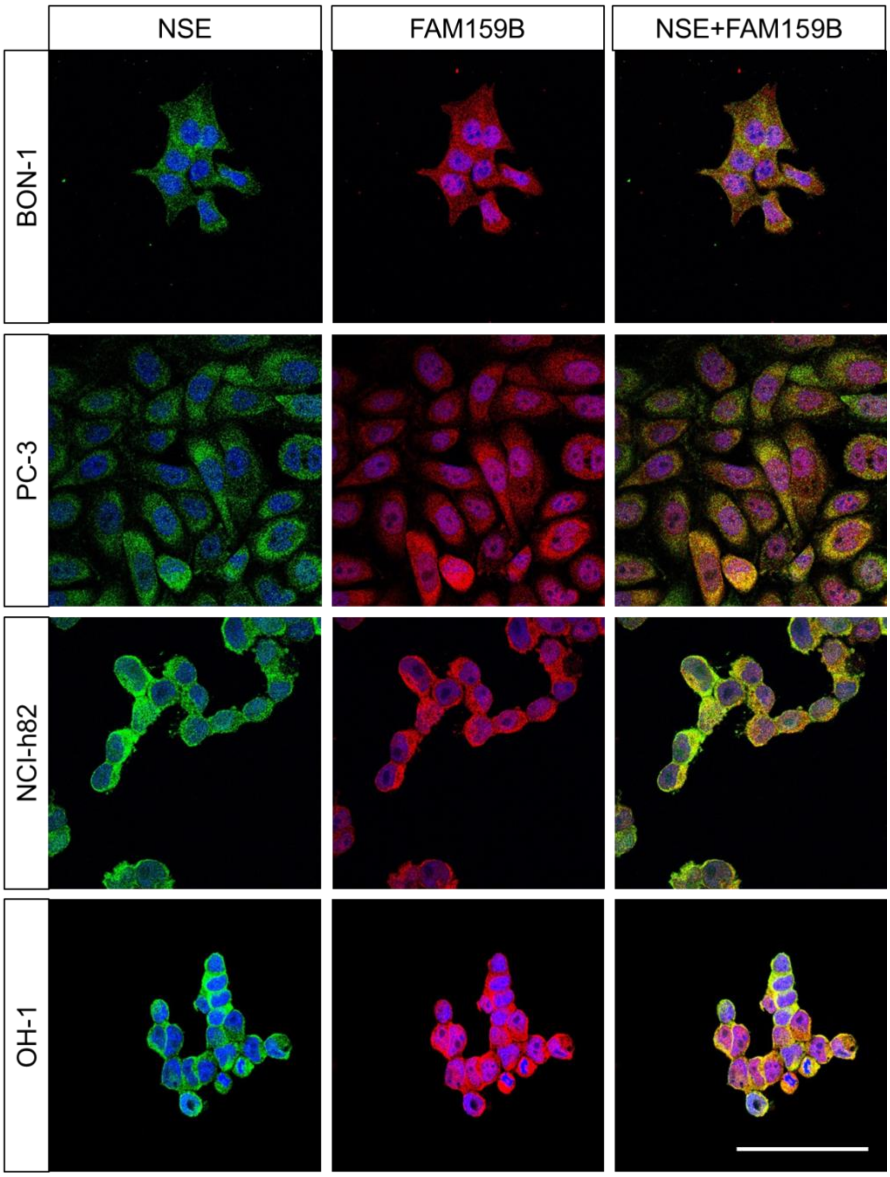


**Supplemental Figure S3: Double-labelling immunocytochemical analysis of FAM159B and neuron-specific enolase (NSE) expression in BON-1, PC-3, NCI-h82, and OH-1 cells.** Labelling for FAM159B was visualised using Cy3-conjugated anti-rabbit antibody (red). Labelling for NSE was visualised using Alexa Fluor 488-conjugated anti-mouse antibody (green). Overlapping expression is shown in orange/yellow colour. All photomicrographs were taken at the same magnification. Scale bar: 100 µm.

## Supplemental Figure S4

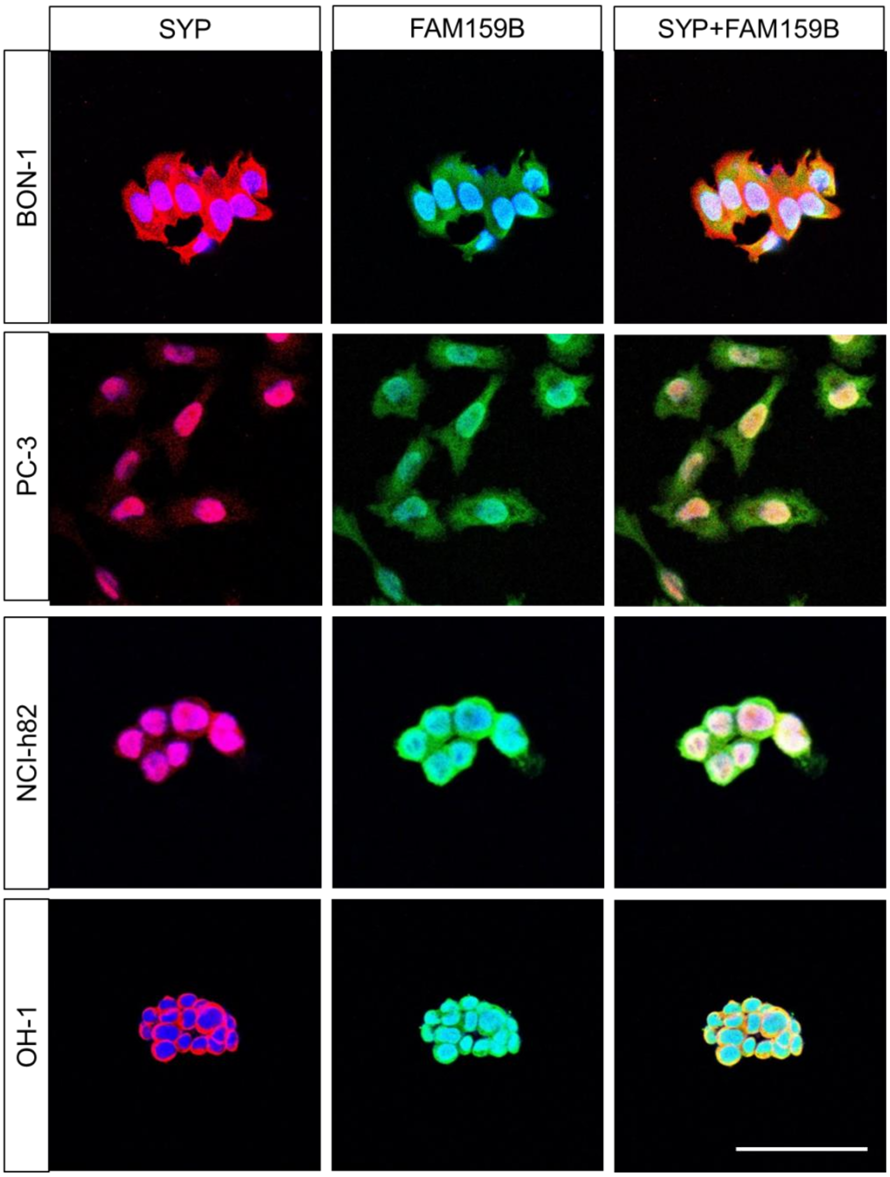


**Supplemental Figure S4: Double-labelling immunocytochemical analysis of FAM159B and synaptophysin (SYP) expression in BON-1, PC-3, NCI-h82, and OH-1 cells.** Labelling for FAM159B was visualised using FITC-conjugated anti-rabbit antibody (green). Labelling for SYP was visualised using Cy3-conjugated anti-rabbit antibody (red). Overlapping expression is shown in orange/yellow colour. All photomicrographs were taken at the same magnification. Scale bar: 100 µm.

## Supplemental Figure S5

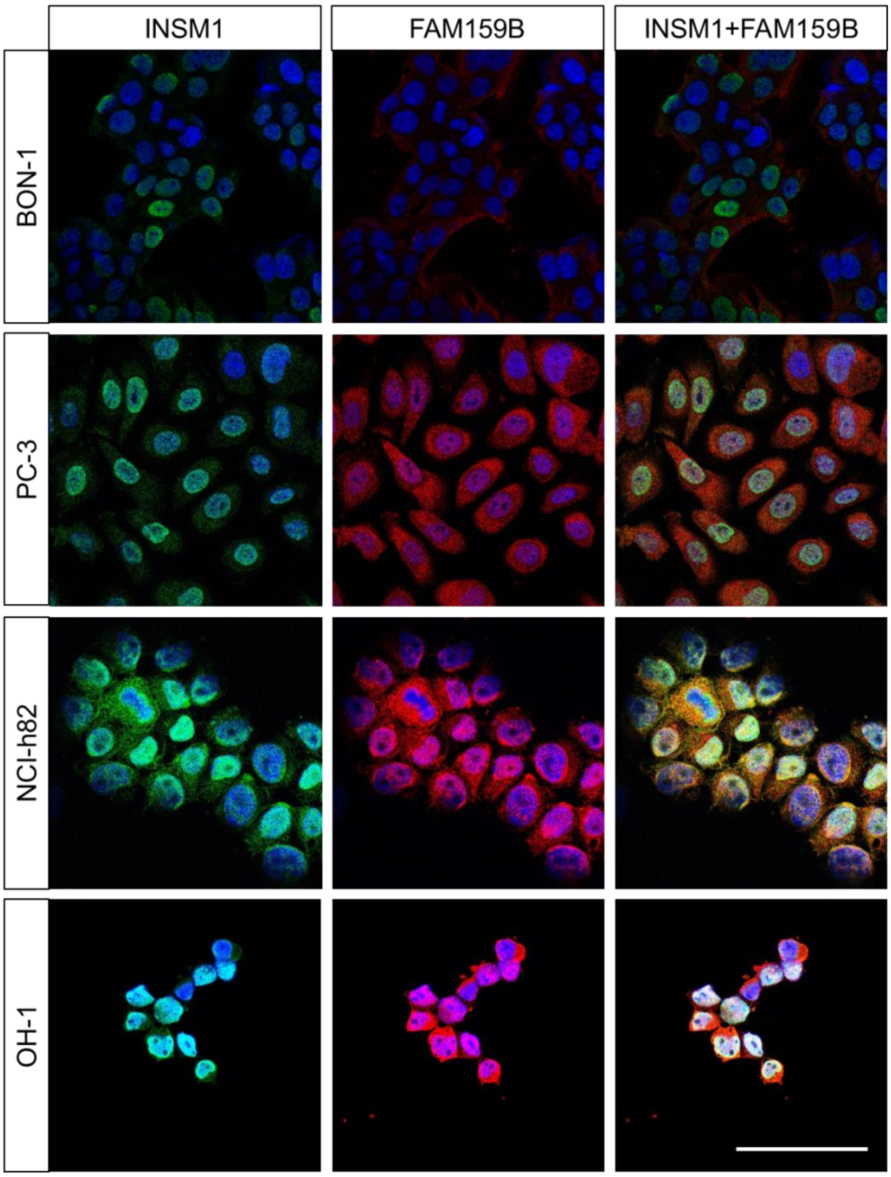


**Supplemental Figure S5: Double-labelling immunocytochemical analysis of FAM159B and insulinoma-associated protein 1 (INSM1) expression in BON-1, PC-3, NCI-h82, and OH-1 cells.** Labelling for FAM159B was visualised using Cy3-conjugated anti-rabbit antibody (red). Labelling for INSM1 was visualised using Alexa Fluor 488-conjugated anti-mouse antibody (green). Overlapping expression is shown in orange/yellow colour. All photomicrographs were taken at the same magnification. Scale bar: 100 µm.

## Supplemental Figure S6

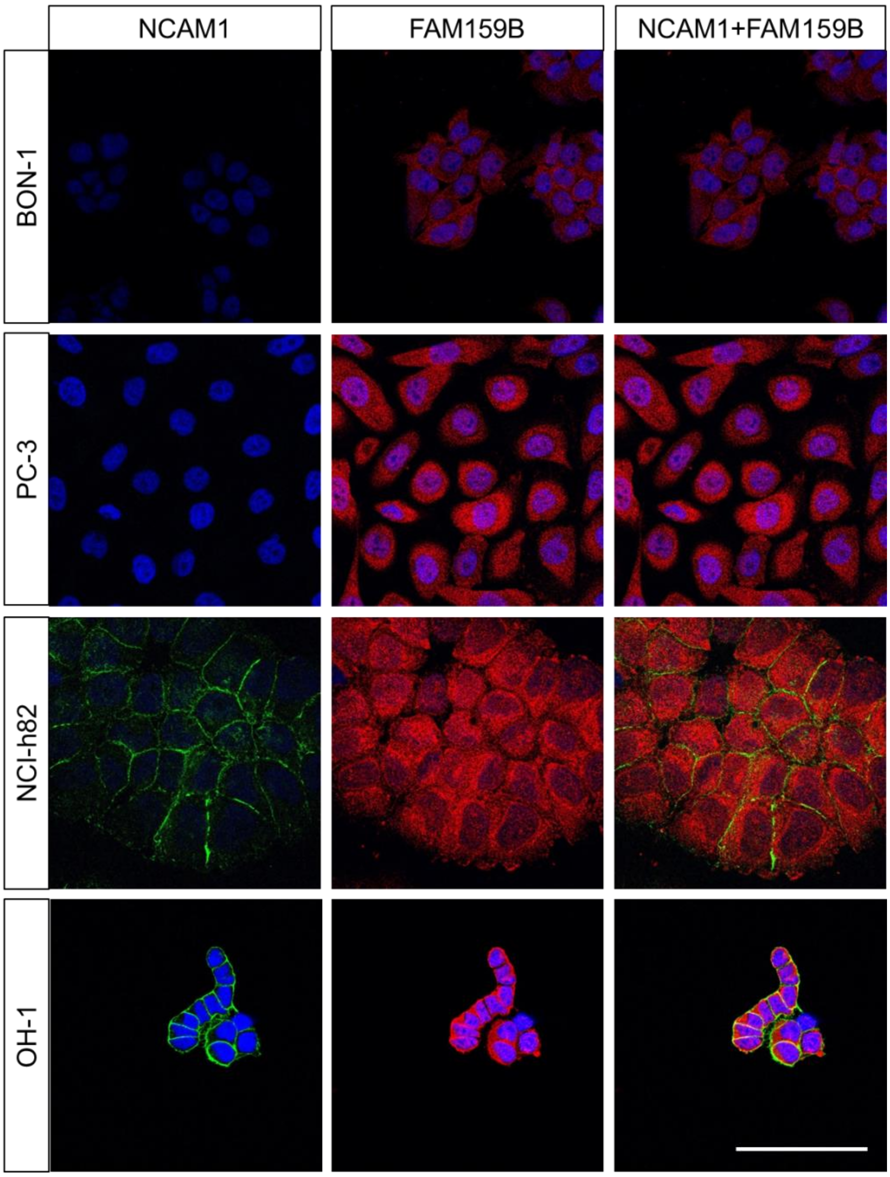


**Supplemental Figure S6: Double-labelling immunocytochemical analysis of FAM159B and neural cell adhesion molecule 1 (NCAM1) expression in BON-1, PC-3, NCI-h82, and OH-1 cells.** Labelling for FAM159B was visualised using Cy3-conjugated anti-rabbit antibody (red). Labelling for NCAM1 was visualised using Alexa Fluor 488-conjugated anti-mouse antibody (green). Overlapping expression is shown in orange/yellow colour. All photomicrographs were taken at the same magnification. Scale bar: 100 µm.

## Supplemental Figure S7

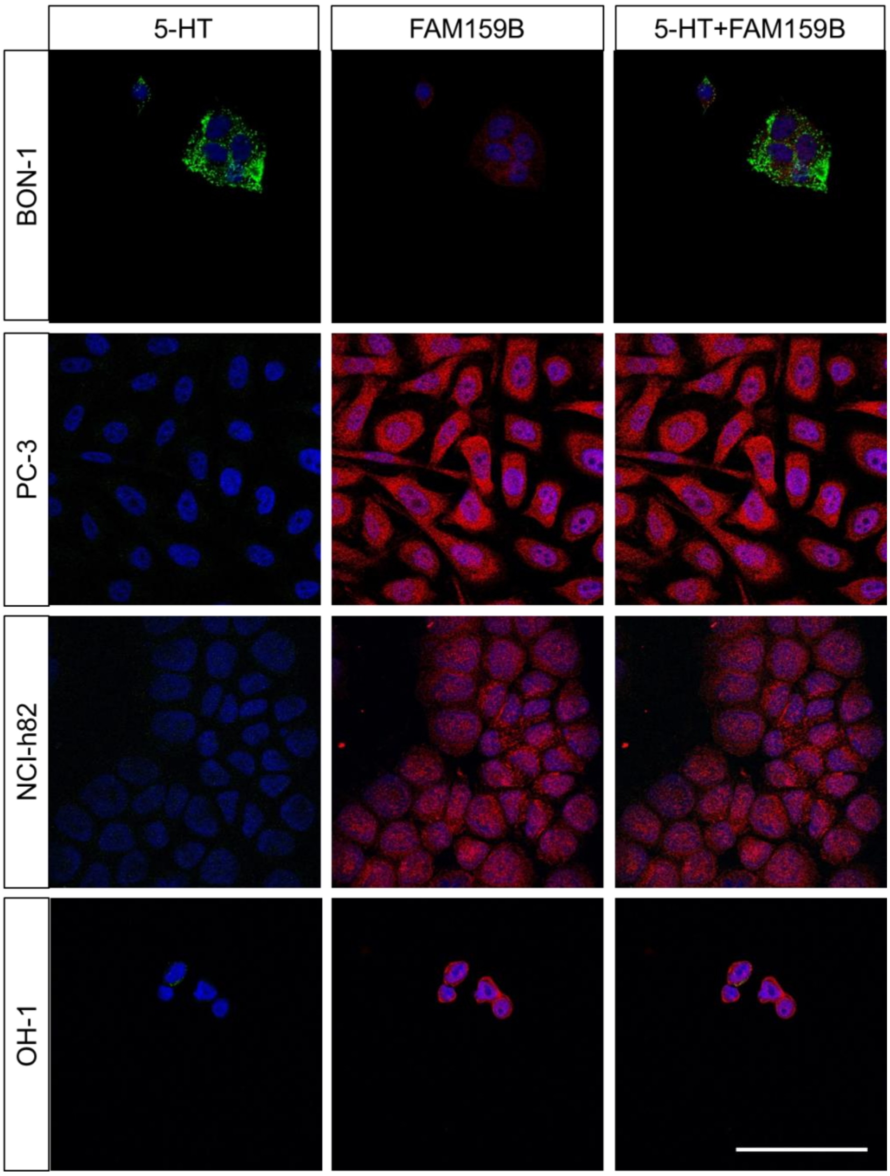


**Supplemental Figure S7: Double-labelling immunocytochemical analysis of FAM159B and serotonin (5-HT) expression in BON-1, PC-3, NCI-h82, and OH-1 cells.** Labelling for FAM159B was visualised using Cy3-conjugated anti-rabbit antibody (red). Labelling for 5-HT was visualised using Alexa Fluor 488-conjugated anti-mouse antibody (green). Overlapping expression is shown in orange/yellow colour. All photomicrographs were taken at the same magnification. Scale bar: 100 µm.

## Supplemental Figure S8

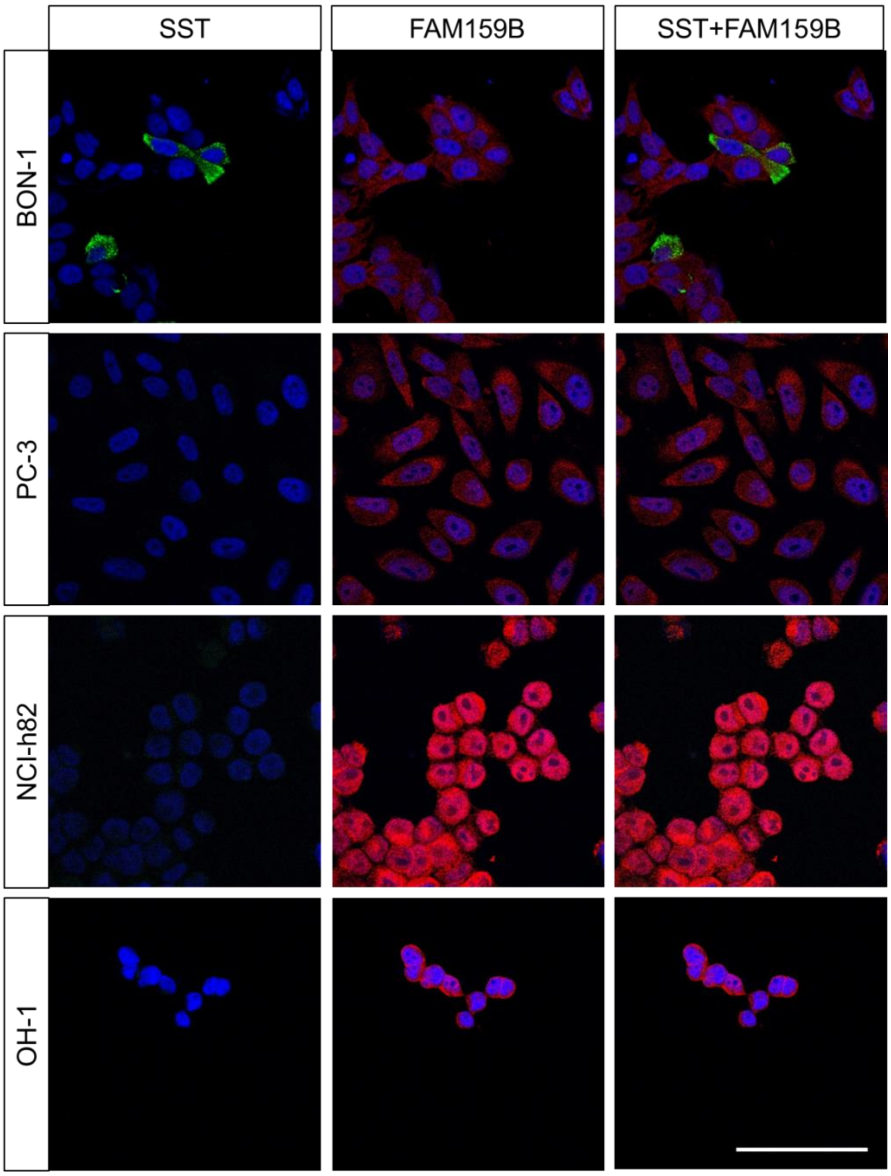


**Supplemental Figure S8: Double-labelling immunocytochemical analysis of FAM159B and somatostatin 14/28 (SST) expression in BON-1, PC-3, NCI-h82, and OH-1 cells.** Labelling for FAM159B was visualised using Cy3-conjugated anti-rabbit antibody (red). Labelling for SST was visualised using Alexa Fluor 488-conjugated anti-rat antibody (green). Overlapping expression is shown in orange/yellow colour. All photomicrographs were taken at the same magnification. Scale bar: 100 µm

## Supplemental Figure S9

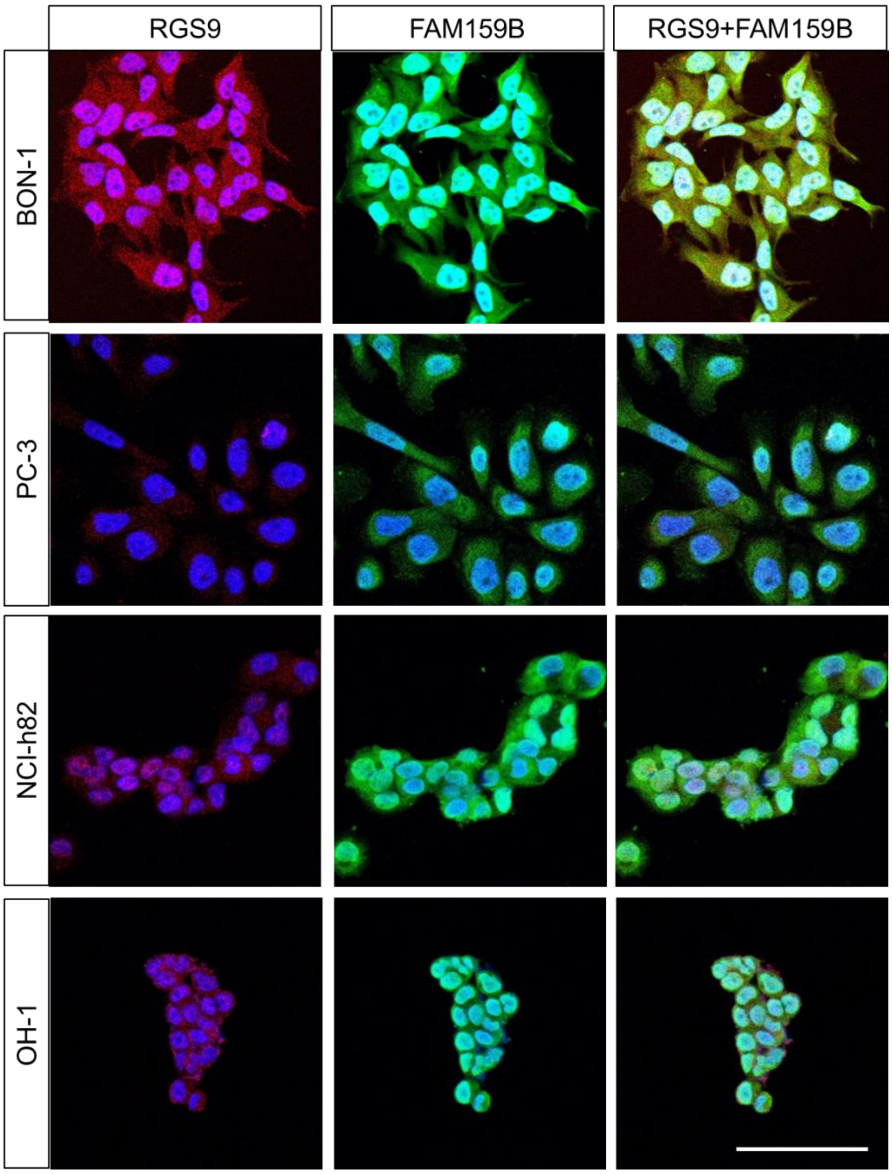


**Supplemental Figure S9: Double-labelling immunocytochemical analysis of FAM159B and regulator of G-protein signalling 9 (RGS9) expression in BON-1, PC-3, NCI-h82, and OH-1 cells.** Labelling for FAM159B was visualised using FITC-conjugated anti-rabbit antibody (green). Labelling for RGS9 was visualised using Cy3-conjugated anti-rabbit antibody (red). Overlapping expression is shown in orange/yellow colour. All photomicrographs were taken at the same magnification. Scale bar: 100 µm.

## Supplemental Figure S10

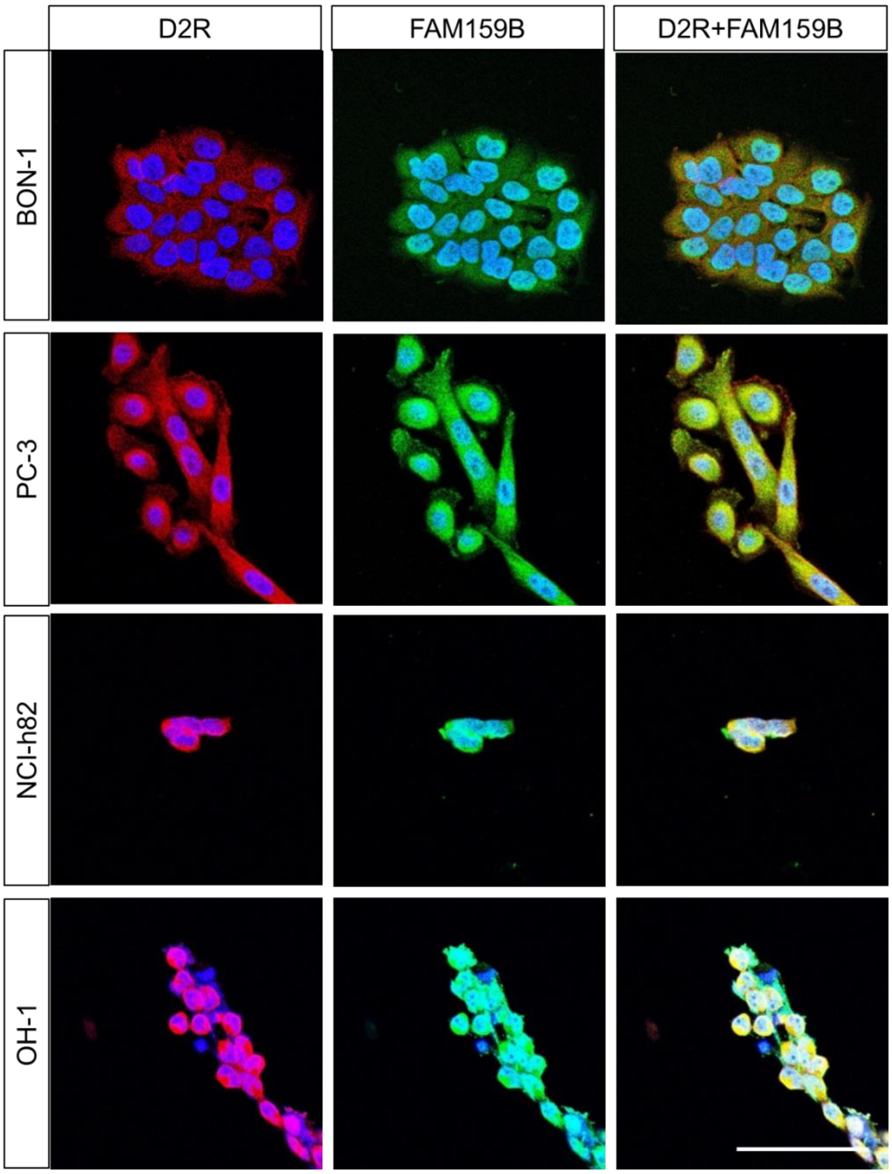


**Supplemental Figure S10: Double-labelling immunocytochemical analysis of FAM159B and dopamine receptor 2 (D2R) expression in BON-1, PC-3, NCI-h82, and OH-1 cells.** Labelling for FAM159B was visualised using FITC-conjugated anti-rabbit antibody (green). Labelling for D2R was visualised using Cy3-conjugated anti-rabbit antibody (red). Overlapping expression is shown in orange/yellow colour. All photomicrographs were taken at the same magnification. Scale bar: 100 µm.

## Supplemental Figure S11

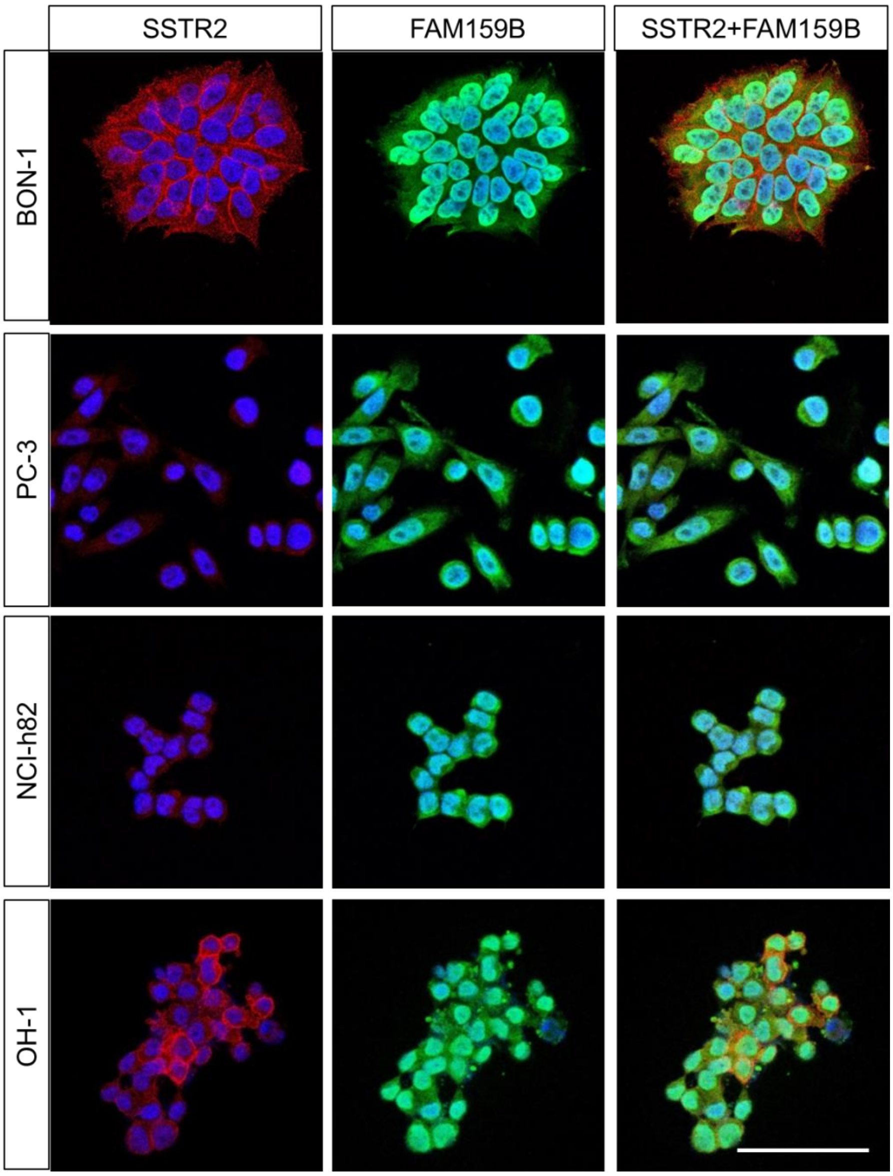


**Supplemental Figure S11: Double-labelling immunocytochemical analysis of FAM159B and somatostatin receptor 2 (SSTR2) expression in BON-1, PC-3, NCI-h82, and OH-1 cells.** Labelling for FAM159B was visualised using FITC-conjugated anti-rabbit antibody (green). Labelling for SSTR2 was visualised using Cy3-conjugated anti-rabbit antibody (red). Overlapping expression is shown in orange/yellow colour. All photomicrographs were taken at the same magnification. Scale bar: 100 µm.

## Supplemental Figure S12

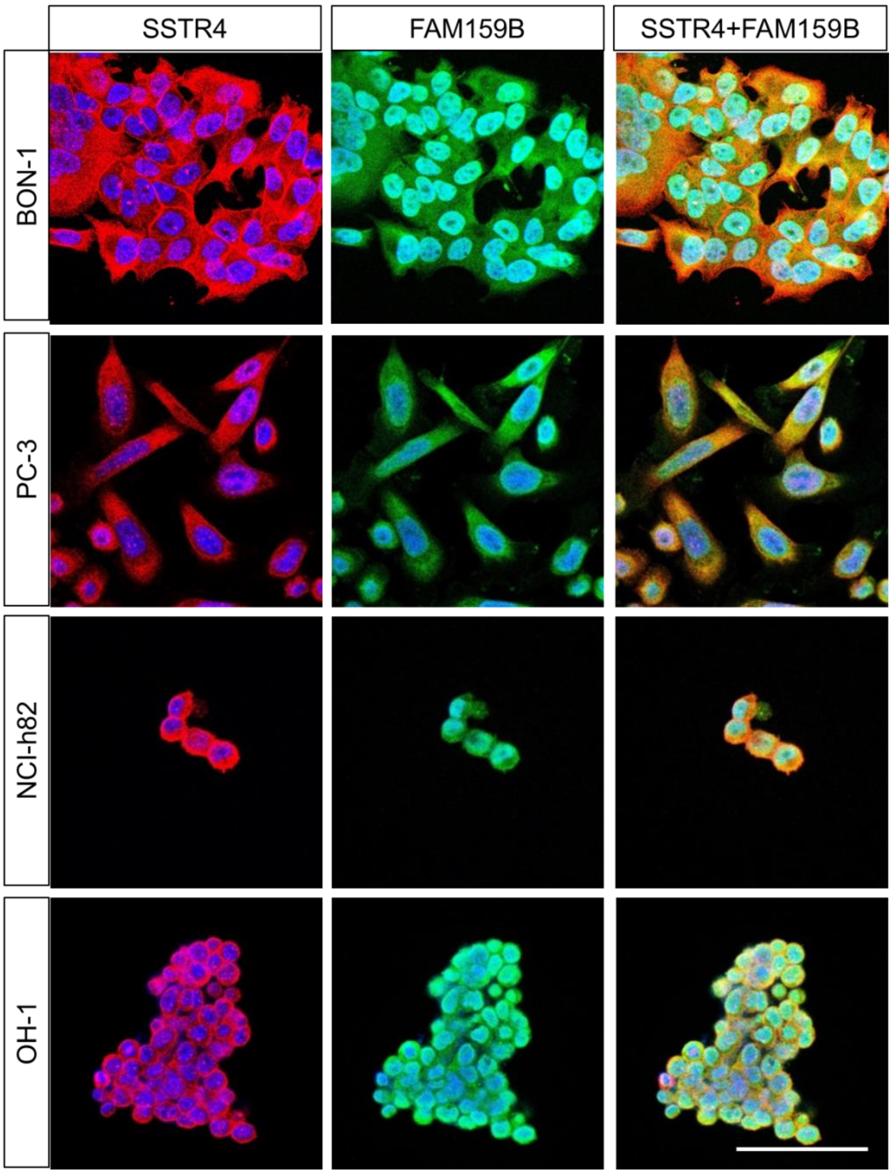


**Supplemental Figure S12: Double-labelling immunocytochemical analysis of FAM159B and somatostatin receptor 4 (SSTR4) expression in BON-1, PC-3, NCI-h82, and OH-1 cells.** Labelling for FAM159B was visualised using FITC-conjugated anti-rabbit antibody (green). Labelling for SSTR4 was visualised using Cy3-conjugated anti-rabbit antibody (red). Overlapping expression is shown in orange/yellow colour. All photomicrographs were taken at the same magnification. Scale bar: 100 µm.

## Supplemental Figure S13

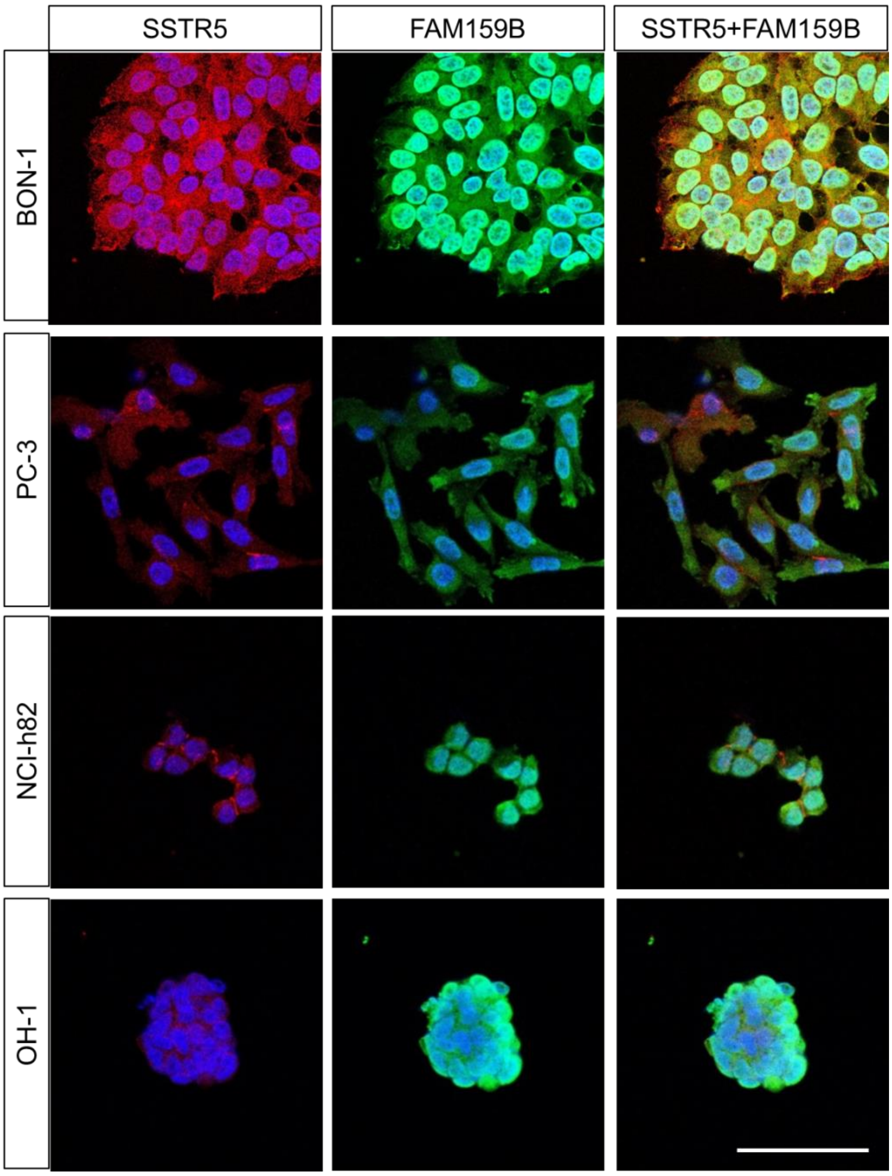


**Supplemental Figure S13: Double-labelling immunocytochemical analysis of FAM159B and somatostatin receptor 5 (SSTR5) expression in BON-1, PC-3, NCI-h82, and OH-1 cells.** Labelling for FAM159B was visualised using FITC-conjugated anti-rabbit antibody (green). Labelling for SSTR5 was visualised using Cy3-conjugated anti-rabbit antibody (red). Overlapping expression is shown in orange/yellow colour. All photomicrographs were taken at the same magnification. Scale bar: 100 µm.

## Supplemental Figure S14

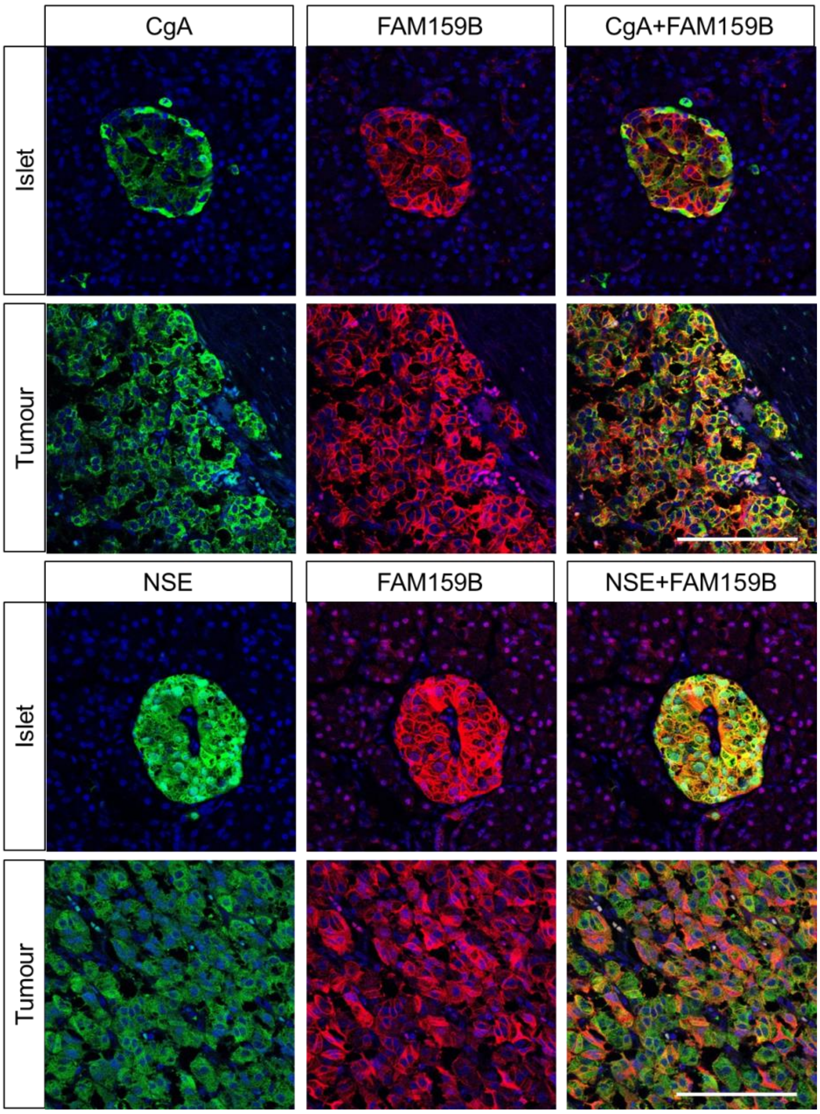


**Supplemental Figure S14: Double-labelling immunohistochemical analysis of FAM159B and chromogranin A (CgA; upper two panels) or neuron-specific enolase (NSE; lower two panels) expression in human pancreas or pancreatic neuroendocrine tumour tissues.** Sections were dewaxed, microwaved in citric acid and incubated with rabbit polyclonal anti-FAM159B antibody HPA011778 and mouse anti-CgA or mouse anti-NSE antibody. Labelling for FAM159B was visualised using Cy3-conjugated anti-rabbit antibody (red). Labelling for CgA or NSE was visualised using Alexa Fluor 488-conjugated anti-mouse antibody (green). Overlapping expression is shown in orange/yellow colour. All photomicrographs were taken at the same magnification. Scale bar: 100 µm.

## Supplemental Figure S15

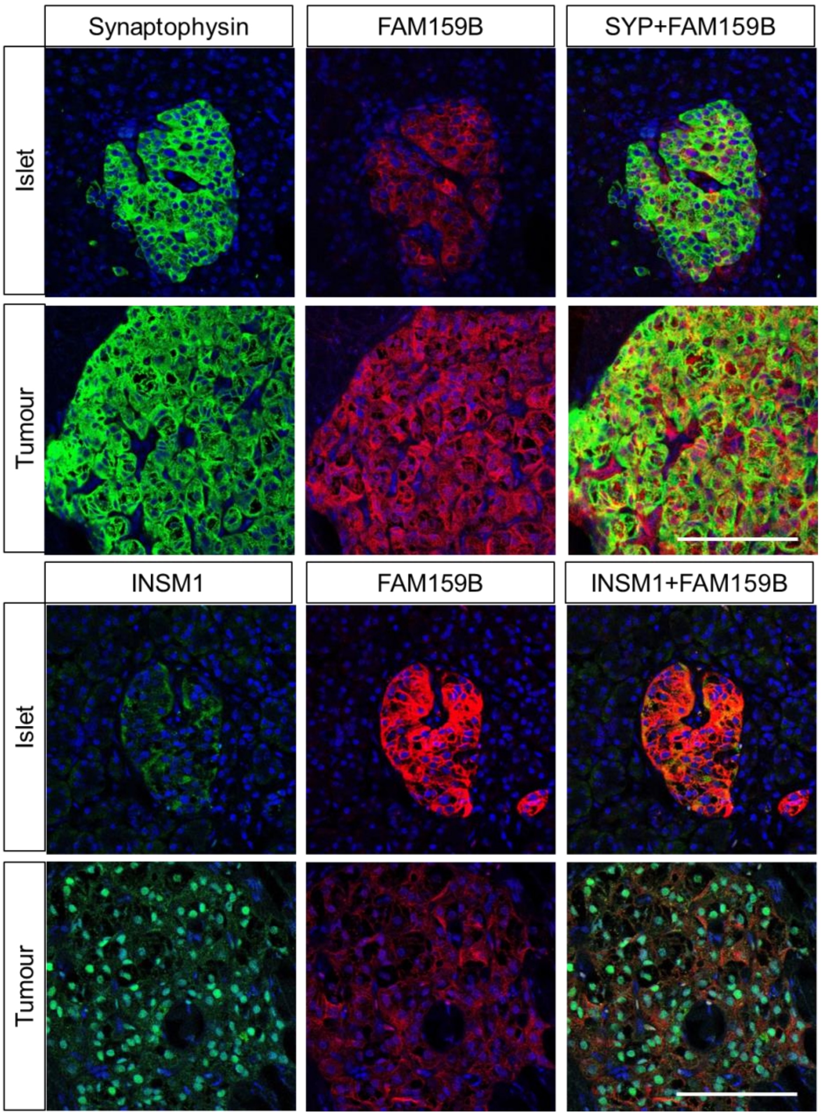


**Supplemental Figure S15: Double-labelling immunohistochemical analysis of FAM159B and synaptophysin (SYP; upper two panels) or insulinoma-associated protein 1 (INSM1; lower two panels) expression in human pancreas or pancreatic neuroendocrine tumour tissues.** For the FAM159B – SYP double-labelling experiments, two separate consecutively cut sections were dewaxed, microwaved in citric acid and incubated either with rabbit polyclonal anti-FAM159B antibody HPA011778 or rabbit anti-SYP antibody. Labelling for FAM159B was visualised using Cy3-conjugated anti-rabbit antibody (red). Labelling for SYP was visualised using Alexa Fluor 488-conjugated anti-rabbit

antibody (green). Finally, two separate images were obtained and then merged digitally. For the FAM159B – INSM1 double-labelling experiments, sections were incubated with rabbit polyclonal anti-FAM159B antibody HPA011778 and mouse anti-INSM1 antibody. Labelling for FAM159B was visualised using Cy3-conjugated anti-rabbit antibody (red). Labelling for INSM1 was visualised using Alexa Fluor 488-conjugated anti-mouse antibody (green). Overlapping expression is shown in orange/yellow colour. All photomicrographs were taken at the same magnification. Scale bar: 100 µm.

## Supplemental Figure S16

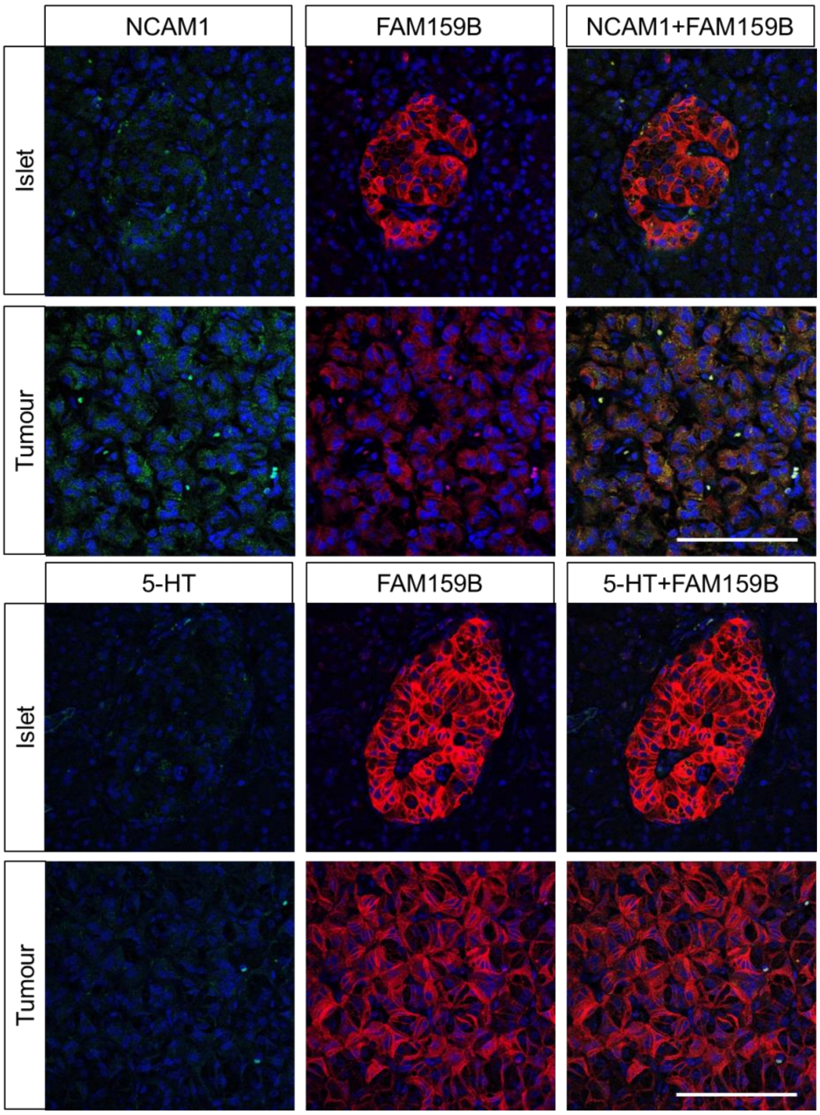


**Supplemental Figure S16: Double-labelling immunohistochemical analysis of FAM159B and neural cell adhesion molecule 1 (NCAM1; upper two panels) or serotonin (5-HT; lower two panels) expression in human pancreas or pancreatic neuroendocrine tumour tissues.** Sections were dewaxed, microwaved in citric acid, and incubated with rabbit polyclonal anti-FAM159B antibody HPA011778 and mouse anti-NCAM1 or mouse anti-5-HT antibody. Labelling for FAM159B was visualised using Cy3-conjugated anti-rabbit antibody (red). Labelling for NCAM1 or 5-HT was visualised using Alexa Fluor 488-conjugated anti-mouse antibody (green). Overlapping expression is shown in orange/yellow colour. All photomicrographs were taken at the same magnification. Scale bar: 100 µm.

## Supplemental Figure S17

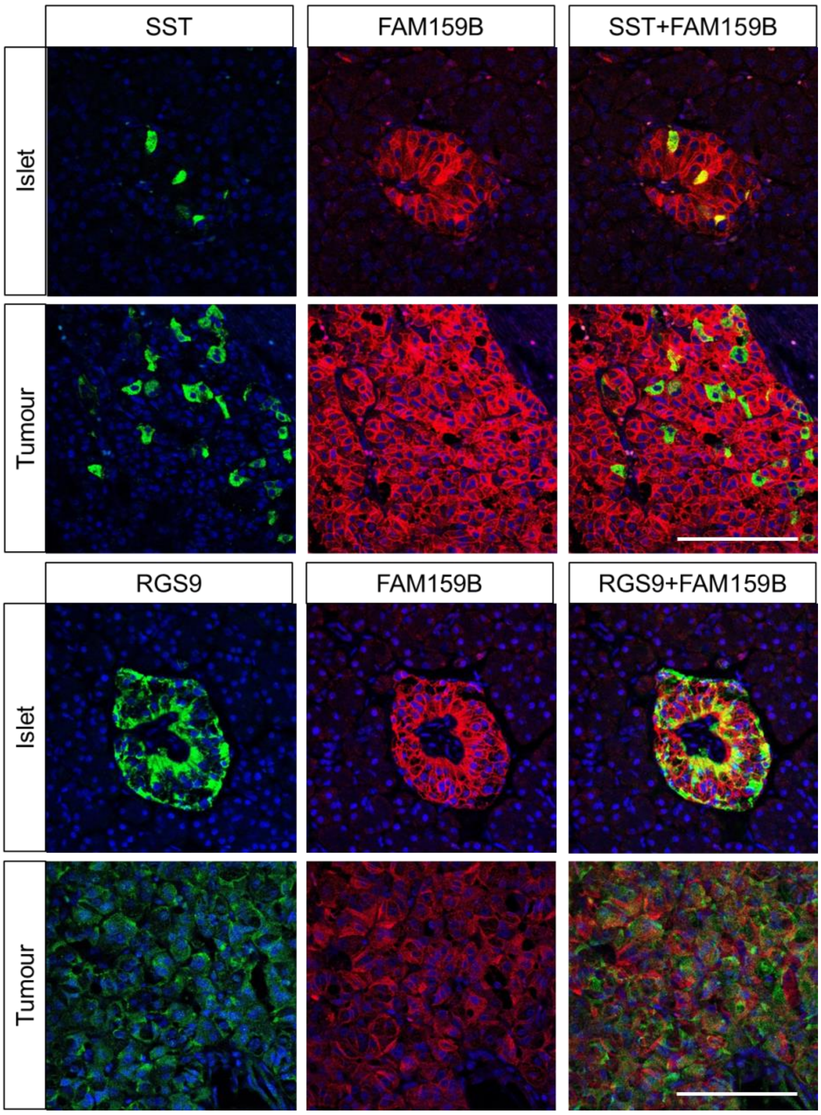


**Supplemental Figure S17: Double-labelling immunohistochemical analysis of FAM159B and somatostatin 14/28 (SST, upper two panels) or regulator of G protein signalling 9 (RGS9; lower two panels) expression in human pancreas or pancreatic neuroendocrine tumour tissues.** For the FAM159B – SST double-labelling experiments, sections were incubated with rabbit polyclonal anti-FAM159B antibody HPA011778 and rat anti-SST antibody. Labelling for FAM159B was visualised using Cy3-conjugated anti-rabbit antibody (red). Labelling for SST was visualised using Alexa Fluor 488-conjugated anti-rat antibody (green). For the FAM159B – RGS9 double-labelling experiments, two separate consecutively cut sections were dewaxed, microwaved in citric acid and incubated either with

rabbit polyclonal anti-FAM159B antibody HPA011778 or rabbit anti-RGS9 antibody. Labelling for FAM159B was visualised using Cy3-conjugated anti-rabbit antibody (red). Labelling for RGS9 was visualised using Alexa Fluor 488-conjugated anti-rabbit antibody (green). Finally, two separate images were obtained and then merged digitally. Overlapping expression is shown in orange/yellow colour. All photomicrographs were taken at the same magnification. Scale bar: 100 µm.

## Supplemental Figure S18

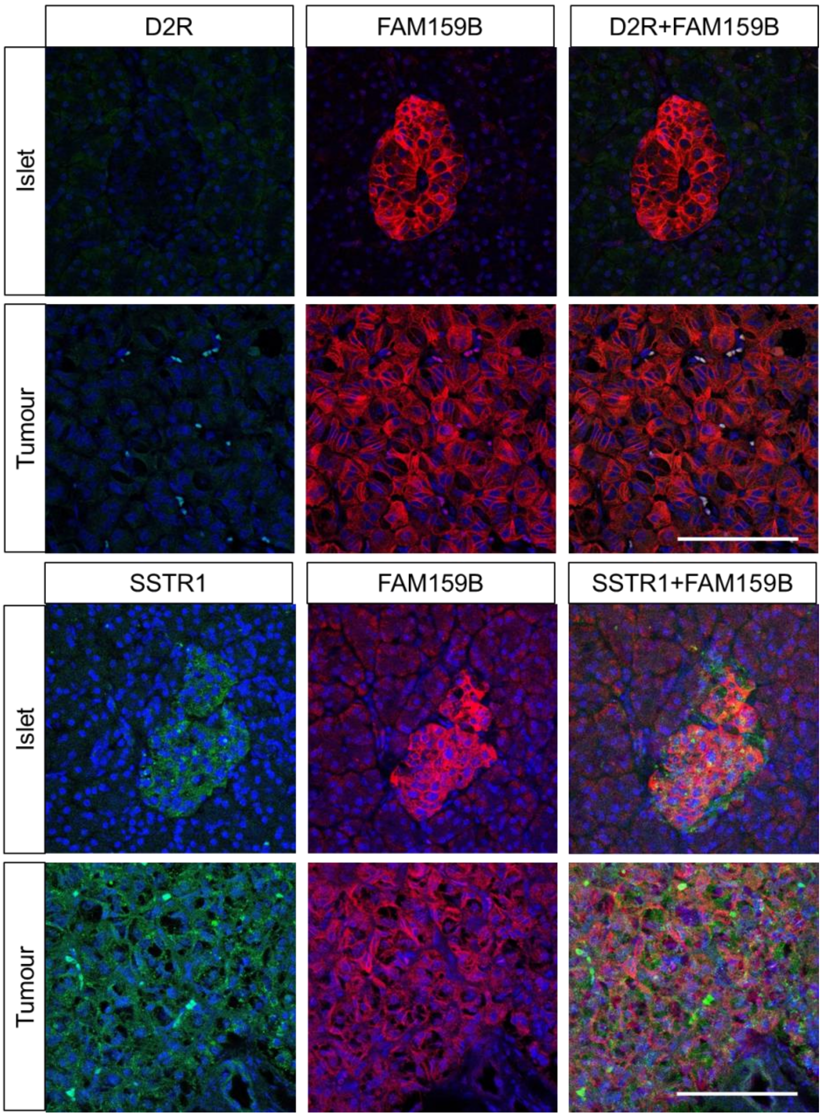


**Supplemental Figure S18: Double-labelling immunohistochemical analysis of FAM159B and dopamine receptor 2 (D2R; upper two panels) or somatostatin receptor 1 (SSTR1; lower two panels) expression in human pancreas or pancreatic neuroendocrine tumour tissues.** For each double-stained image, two separate consecutively cut sections were dewaxed, microwaved in citric acid, and incubated either with rabbit polyclonal anti-FAM159B antibody HPA011778 or rabbit anti-D2R antibody (upper two panels), respectively, or with rabbit polyclonal anti-FAM159B antibody HPA011778 or rabbit anti-SSTR1 antibody (lower two panels), respectively. Labelling for FAM159B was visualised using Cy3-conjugated anti-rabbit antibody (red). Labelling for D2R and SSTR1 was visualised using

Alexa Fluor 488-conjugated anti-rabbit antibody (green). In each case, two separate images were obtained and then merged digitally. Overlapping expression is shown in orange/yellow colour. All photomicrographs were taken at the same magnification. Scale bar: 100 µm.

## Supplemental Figure S19

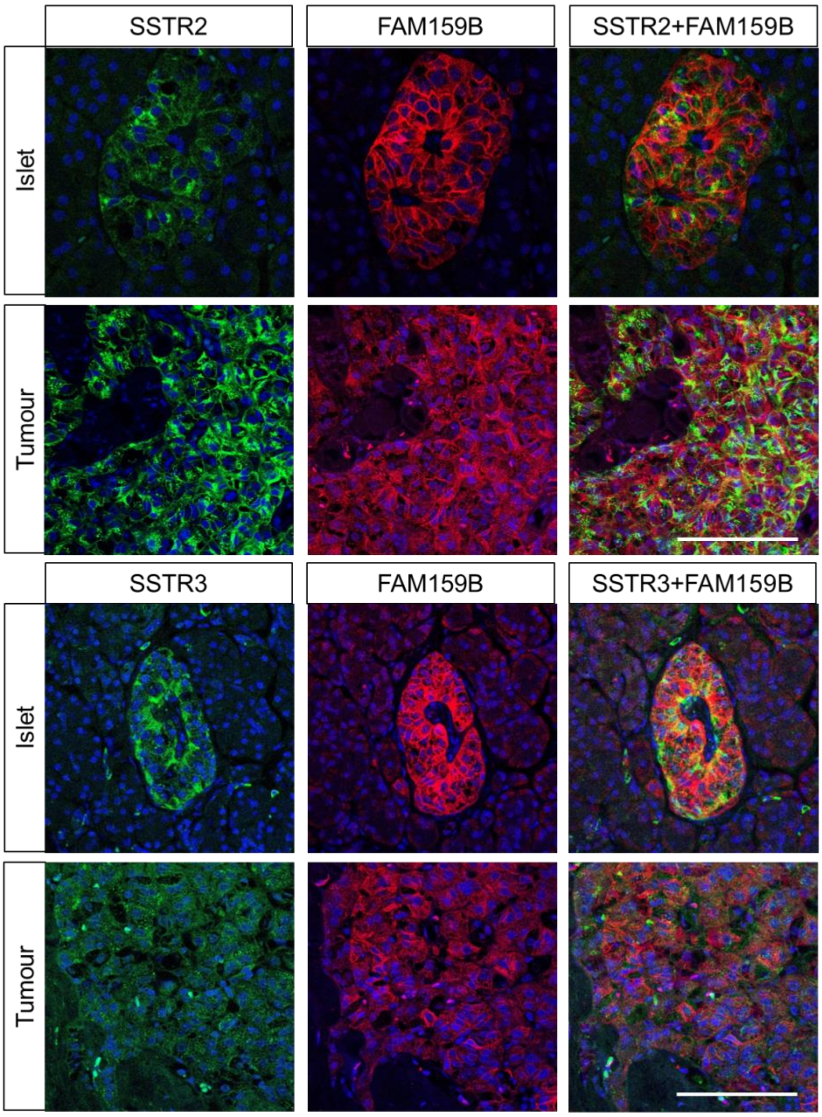


**Supplemental Figure S19: Double-labelling immunohistochemical analysis of FAM159B and somatostatin receptor 2 (SSTR2; upper two panels) or somatostatin receptor 3 (SSTR3; lower two panels) expression in human pancreas or pancreatic neuroendocrine tumour tissues.** For each double-stained image, two separate consecutively cut sections were dewaxed, microwaved in citric acid, and incubated with rabbit polyclonal anti-FAM159B antibody HPA011778 or rabbit anti-SSTR1 antibody (upper two panels), respectively, or with rabbit polyclonal anti-FAM159B antibody HPA011778 or rabbit anti-SSTR2 antibody (lower two panels), respectively. Labelling for FAM159B was visualised using Cy3-conjugated anti-rabbit antibody (red). Labelling for SSTR3 and SSTR3

was visualised using Alexa Fluor 488-conjugated anti-rabbit antibody (green). In each case, two separate images were obtained and then merged digitally. Overlapping expression is shown in orange/yellow colour. All photomicrographs were taken at the same magnification. Scale bar: 100 µm.

## Supplemental Figure S20

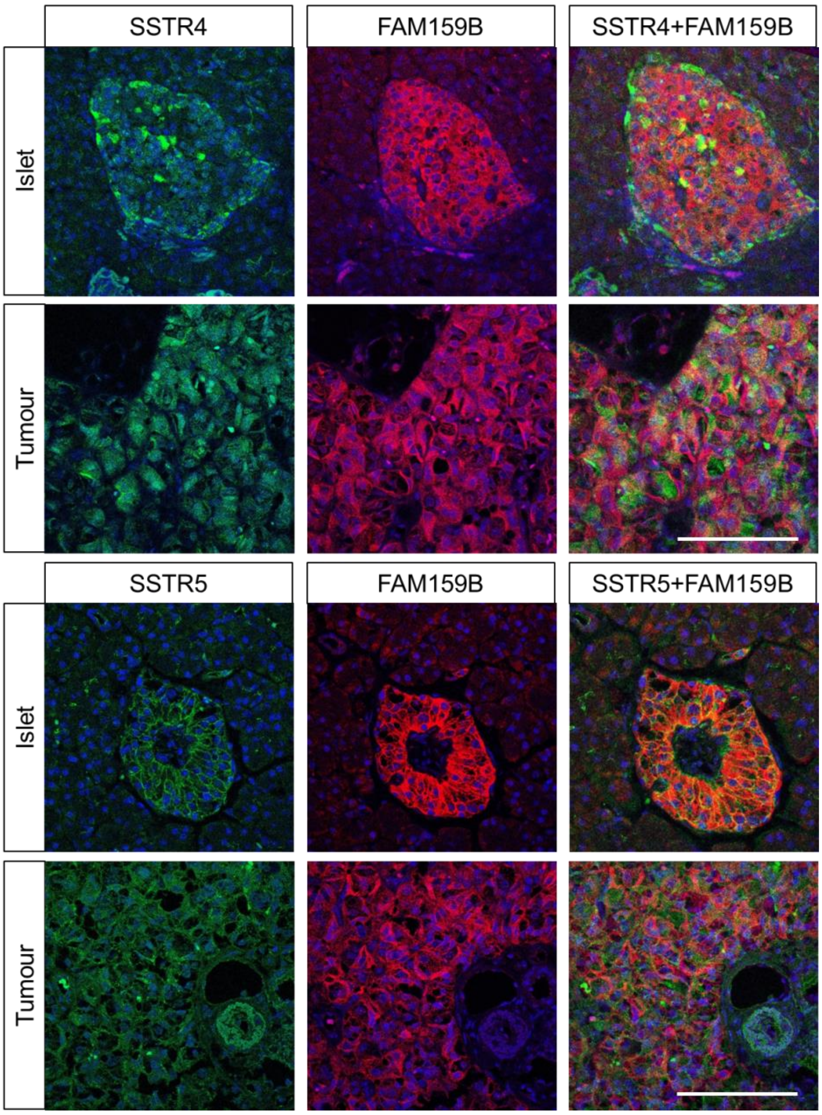


**Supplemental Figure S20: Double-labelling immunohistochemical analysis of FAM159B and somatostatin receptor 4 (SSTR4; upper two panels) or somatostatin receptor 5 (SSTR5; lower two panels) expression in human pancreas or pancreatic neuroendocrine tumour tissues.** For each double-stained image, two separate consecutively cut sections were dewaxed, microwaved in citric acid, and incubated with rabbit polyclonal anti-FAM159B antibody HPA011778 or rabbit anti-SSTR4 antibody (upper two panels), respectively, or with rabbit polyclonal anti-FAM159B antibody HPA011778 or rabbit anti-SSTR5 antibody (lower two panels), respectively. Labelling for FAM159B was visualised using Cy3-conjugated anti-rabbit antibody (red). Labelling for SSTR4 and SSTR5

was visualised using Alexa Fluor 488-conjugated anti-rabbit antibody (green). In each case, two separate images were obtained and then merged digitally. Overlapping expression is shown in orange/yellow colour. All photomicrographs were taken at the same magnification. Scale bar: 100 µm.
